# Characterization of the dispirotripiperazine derivative PDSTP as antibiotic adjuvant and antivirulence compound against *Pseudomonas aeruginosa*

**DOI:** 10.3389/fmicb.2024.1357708

**Published:** 2024-02-16

**Authors:** Andrea Bonacorsi, Gabriele Trespidi, Viola C. Scoffone, Samuele Irudal, Giulia Barbieri, Olga Riabova, Natalia Monakhova, Vadim Makarov, Silvia Buroni

**Affiliations:** ^1^Department of Biology and Biotechnology "Lazzaro Spallanzani", University of Pavia, Pavia, Italy; ^2^Research Center of Biotechnology RAS, Moscow, Russia

**Keywords:** *Pseudomonas aeruginosa*, drug resistance, antibiotic adjuvant, combination therapy, antivirulence, antiadhesion, antibiofilm

## Abstract

*Pseudomonas aeruginosa* is a major human pathogen, able to establish difficult-to-treat infections in immunocompromised and people with cystic fibrosis (CF). The high rate of antibiotic treatment failure is due to its notorious drug resistance, often mediated by the formation of persistent biofilms. Alternative strategies, capable of overcoming *P. aeruginosa* resistance, include antivirulence compounds which impair bacterial pathogenesis without exerting a strong selective pressure, and the use of antimicrobial adjuvants that can resensitize drug-resistant bacteria to specific antibiotics. In this work, the dispirotripiperazine derivative PDSTP, already studied as antiviral, was characterized for its activity against *P. aeruginosa* adhesion to epithelial cells, its antibiotic adjuvant ability and its biofilm inhibitory potential. PDSTP was effective in impairing the adhesion of *P. aeruginosa* to various immortalized cell lines. Moreover, the combination of clinically relevant antibiotics with the compound led to a remarkable enhancement of the antibiotic efficacy towards multidrug-resistant *CF* clinical strains. PDSTP-ceftazidime combination maintained its efficacy *in vivo* in a *Galleria mellonella* infection model. Finally, the compound showed a promising biofilm inhibitory activity at low concentrations when tested both *in vitro* and using an *ex vivo* pig lung model. Altogether, these results validate PDSTP as a promising compound, combining the ability to decrease *P. aeruginosa* virulence by impairing its adhesion and biofilm formation, with the capability to increase antibiotic efficacy against antibiotic resistant strains.

## 1 Introduction

Epidemiological studies have shown that about 1.27 million people died from infections caused by antibiotic-resistant bacteria in 2019 (Antimicrobial Resistance Collaborators, 2022), a number that could grow to 10 million by 2050 (de Kraker et al., 2016). Among these threatening bacteria, *Pseudomonas aeruginosa* plays an important role, especially when carbapenem-resistant strains are involved. Indeed, this bacterium is listed among the “critical” pathogens by the World Health Organization (Tacconelli et al., 2018).

*P. aeruginosa* is involved in upper and lower airway infections in people with cystic fibrosis (CF) patients, that ultimately lead to respiratory failure (Qin et al., 2022). This is particularly relevant as the prevalence of this pathogen in CF adults can reach 80–90% in some European countries (Orenti et al., 2022). Also, this bacterium belongs to the ESKAPE pathogens and is generally associated with nosocomial infections characterized by a broad antibiotic resistance (Qin et al., 2022).

Having a large versatile genome, *P. aeruginosa* can adapt to several hostile niches within the human body, being also equipped with many virulence factors (Qin et al., 2022). One of the main challenges in the treatment of this pathogen is connected to its ability to form biofilms, generally associated with the establishment of chronic infections difficult to eradicate: it is estimated that bacteria within biofilms can exhibit a 10–1,000-fold increased antibiotic resistance compared to their planktonic form (Tuon et al., 2022).

Currently, the treatment of *P. aeruginosa* infections primarily involves the administration of ceftazidime, ciprofloxacin, tobramycin and colistin (Wood et al., 2023). However, these compounds are becoming ineffective because of intrinsic, acquired and adaptive antibiotic resistance.

Given the limited progress in the development of new antibiotics and the threat of infections with emerging antibiotic-resistant strains, there is an urgent need to develop alternative therapeutic strategies, including the repurposing of existing drugs (Panda et al., 2022; Sanya et al., 2023). Among the various approaches, antivirulence strategies are particularly promising due to their ability to impair bacterial pathogenesis, without killing the bacteria, so exerting lower selective pressure and reducing resistance development compared to conventional antibiotics (Liao et al., 2022). Moreover, it is worth considering combination therapies which involve the use of existing and novel antimicrobials with compounds that increase membrane permeability, inhibit efflux pumps or impair signaling pathways connected to antibiotic resistance (Wang et al., 2020).

**Figure 1:**
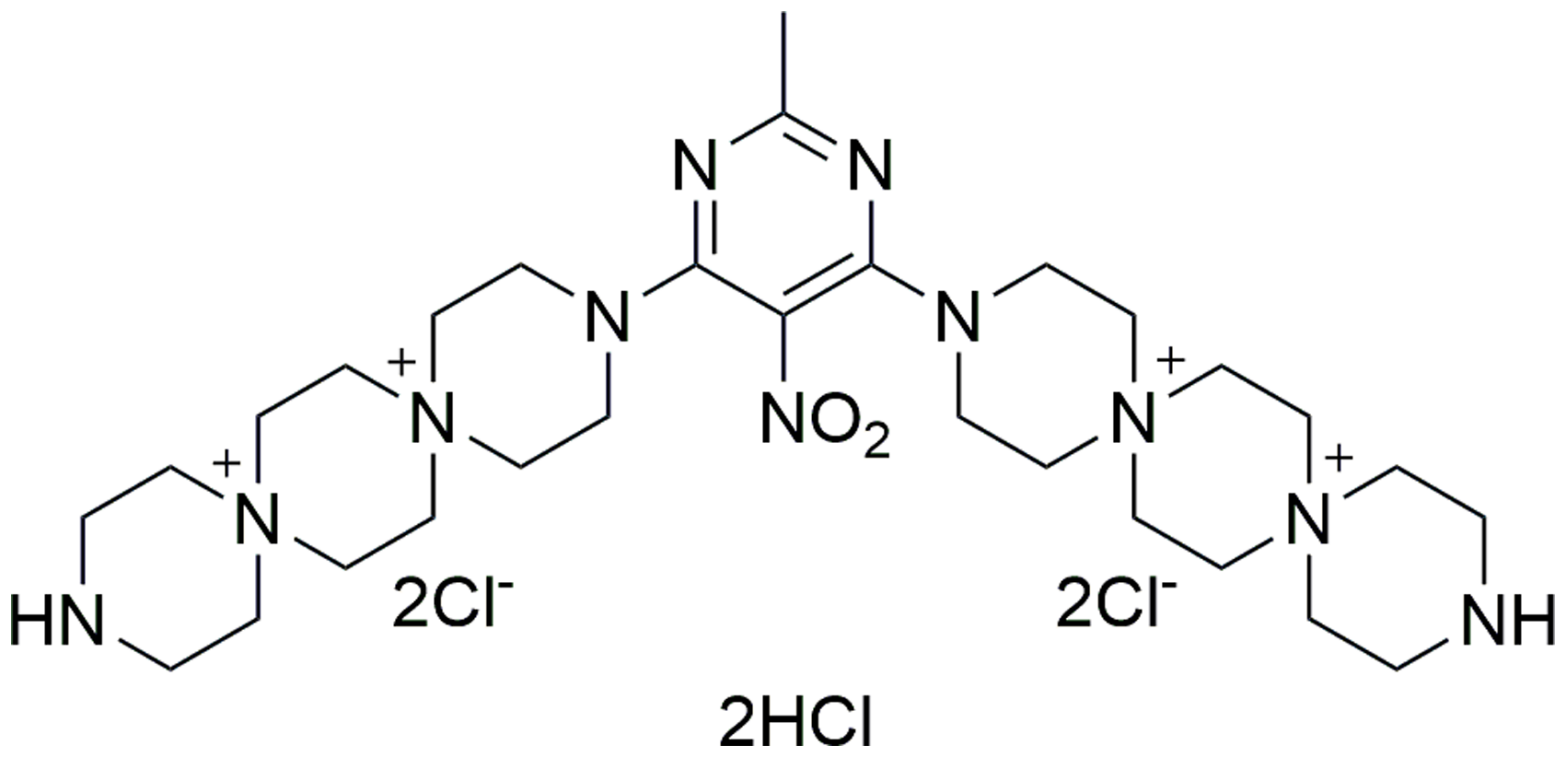
Chemical structure of the compound PDSTP.

PDSTP is a non-toxic dispirotripiperazine-based compound characterized by four quaternary positively charged nitrogen atoms (Figure 1). It has been investigated as antiviral since it impairs viral adsorption to negatively charged heparan-sulphate glycosaminoglycans (HSGAGs) expressed at the surface of human cells by saturating them through electrostatic interactions (Schmidtke et al., 2002). In particular, PDSTP exhibits a good antiviral activity *in vitro* when tested on herpes simplex virus type 1 (Schmidtke et al., 2002) and on human immunodeficiency virus type 1 and 2 (Novoselova et al., 2017). In addition, this compound prevents the death of a herpetic encephalitis mouse model when utilized in combination with acyclovir (Novoselova et al., 2019), is more effective compared to acyclovir when used to treat a guinea pig model of genital herpes (Novoselova et al., 2020), reduces both corneal lesions and viral infection course in a rabbit model of herpes simplex epithelial keratitis (Alimbarova et al., 2022) and prevents severe viral pneumonia induced by SARS-CoV-2 in a Syrian hamster infection model (Makarov and Popov, 2022).

Besides viruses, bacteria have evolved to use HSGAGs as receptors (García et al., 2016). Specifically, *P. aeruginosa* interacts with HS chains through bacterial adhesins (Bucior et al., 2012). The probability of this interaction increases when epithelia are injured: for instance, when the respiratory epithelium is damaged, polarized epithelial cells dedifferentiate and increase the expression of HS proteoglycans at their apical surface, promoting bacterial adhesion. In this context, PDSTP may be active not only on viruses, but also on bacteria, preventing their interaction with HSGAGs and, possibly, their adhesion to human cells.

The aim of this work was to investigate the putative anti-adhesive properties of PDSTP on *P. aeruginosa* by adhesion assay and imaging flow cytometry, testing the compound on different human cell lines challenged with *P. aeruginosa* clinical isolates. In addition, antimicrobial combination susceptibility testing and time-killing assays were performed on a panel of *P. aeruginosa* clinical isolates to determine whether PDSTP potentiates the activity of antibiotics currently utilized in clinics. Antibiotic potentiation was then evaluated *in vivo* employing a *Galleria mellonella* infection model. Finally, the ability of the compound to impair biofilm formation was determined both *in vitro* and *ex vivo*.

## 2 Materials and methods

### 2.1 Bacterial strains, growth conditions, antibiotics, and compounds

The *P. aeruginosa* reference strain PA01 (laboratory collection) and 9 *P. aeruginosa* CF clinical isolates (BST44, SG2, NN2, NN83, NN84, RP73, RP74, BT2, and BT72) (Alcalá-Franco et al., 2012) were grown in tryptic soy broth (TSB; BD) or cation-adjusted Mueller-Hinton broth (MHB; BD) at 37°C. The synthetic cystic fibrosis sputum medium (SCFM) was prepared with 0.1% casamino acids (Harrington et al., 2021).

PDSTP, short for 3,3′-(2-methyl-5-nitropyrimidine-4,6-diyl)bis-3,12-diaza-6,9-diazonia-dispiro (5.2.5.2) hexadecane tetrachloride dihydrochloride nonahydrate, was synthesized at the Research Centre of Biotechnology RAS (Moscow, Russia) as described previously (Schmidtke et al., 2002) and its purity was determined by high-performance liquid chromatography analysis (Supplementary Figure S8).

Tested antibiotics were amikacin (Sigma-Aldrich), ceftazidime (Sigma-Aldrich), ciprofloxacin (Sigma-Aldrich), colistin (Sigma-Aldrich), meropenem (Venus Pharma GmbH) and tobramycin (Sigma-Aldrich); ciprofloxacin was dissolved in 0.1 N NaOH, while the other antibiotics were dissolved in water. PDSTP and heparin (Sigma-Aldrich) were dissolved in water.

### 2.2 Human pulmonary epithelial cell cultures

Human pulmonary epithelial cells were routinely cultured in 75 cm^2^ flasks either in Dulbecco’s modified Eagle’s medium (DMEM; Euroclone) supplemented with 10% foetal bovine serum (FBS; Euroclone), 0.1 mM MEM non-essential amino acids (Euroclone), 100 U/mL penicillin and 0.1 mg/mL streptomycin (Euroclone) or minimal essential medium (MEM; Euroclone) supplemented with 10% FBS, 2 mM glutamine (Euroclone), 100 U/mL penicillin and 0.1 mg/mL streptomycin, at 37°C in 5% CO_2_. Specifically, A549 cells (Giard et al., 1973) were cultured in DMEM, while 16HBE14o- (Cozens et al., 1994) and CFBE41o-, carrying the biallelic ΔF508 mutation (Bruscia et al., 2002), were grown in MEM.

### 2.3 Quantification of bacterial adhesion

#### 2.3.1 Quantification of the adhesion by plate counting

Adhesion assays by plating adhered bacteria were performed as previously described (Berlutti et al., 2008), with appropriate modifications. Briefly, 1.5 × 10^5^ cells per well were seeded in 24-well tissue culture plates and cultured in medium without antibiotics for 48 h. Two-day old confluent monolayers were infected with bacteria collected during the mid-log phase of growth and resuspended in medium without antibiotics at a multiplicity of infection (MOI) of 10 bacteria per human cell. At the time of the infection, PDSTP and/or heparin were added at the desired concentration in duplicate, while two wells were not treated (control wells). After 2 h of incubation at 4°C (Berlutti et al., 2008), cell monolayers were gently washed three times with phosphate-buffered saline (PBS; Sigma-Aldrich) to remove non-adherent bacteria, lysed with 1% Triton (Riedel-de Haën) and appropriate dilutions of the cell lysates were plated to enumerate adhered bacteria. The percentage of bacterial adhesion to human cells was calculated as:


$$\frac{\mathrm{Number}\;\mathrm{of}\;\mathrm{CFUs}\;\mathrm{of}\;\mathrm{adhered}\;\mathrm{bacteria}}{\mathrm{Number}\;\mathrm{of}\;\mathrm{CFUs}\;\mathrm{of}\;\mathrm{inoculated}\;\mathrm{bacteria}}\;x\;100$$

The percentage of bacterial adhesion was then normalized on the untreated sample, set at 100%.

#### 2.3.2 Quantification of the adhesion by imagestream flow cytometry analysis

After incubating the infected monolayers (MOI = 100) with an isogenic *P. aeruginosa* PA01 strain constitutively expressing GFP (Crabbé et al., 2017) for 2 h at 4°C and washing them four times with ice-cold PBS to remove non-adherent bacteria, quantification of the adhesion by ImageStream Flow Cytometry was performed as follows. Human cells were gently detached from the 24-well tissue culture plate with a cell scraper, washed by centrifugation at 4°C with 1 mL of Dulbecco’s PBS (Sigma-Aldrich) and resuspended in 50 μL of the same buffer to obtain a final concentration of 2 × 10^7^ cells/mL. Samples were kept on ice until the analysis at the Amnis ImageStreamX MkII instrument (Cytek).

For each sample, 10.000 events were acquired at 40X magnification (NA = 0.75; core size = 10 μm) with 488 nm laser excitation (100 mW). Brightfield images were collected in channel 4, cell-bacteria complexes in channel 2 (480–560 nm channel width, 528/65 bandpass) and channel 6 (745–800 nm width, 762/35 bandpass) was used for scatterplot (SSC) detection. Sheath fluid without Mg^2+^ and Ca^2+^ (D-PBS, ThermoFisher) was used in all measurements.

Acquisition was performed by Inspire software (Amnis, version 1.3) with the following gating strategy: focused cells linear gate G1 (GradientRMS_Ch04) and selecting single cells square gate G2 (AspectRatio_Ch04/Area_Ch04).

Data analysis was made using Amnis IDEAS software (version 6.2): focused cells gate (“GradientRMS_Ch04” feature), single cells gate (“AspectRatio_Ch04”/“Area_Ch04” features) and finally the custom “BactCount” features were applied for quantification (Supplementary Figure S2). “BactCount” feature was created as follows: a custom “BactCount” mask was created (PeakM02,Ch02,Bright24.5) and the “SpotCount” feature was applied on this mask.

### 2.4 Minimum inhibitory concentration (MIC) determination of PDSTP alone and in combination with antibiotics

The MICs of PDSTP alone and in combination with antibiotics were determined in MHB using the broth microdilution method, according to the EUCAST guidelines (Mann and Markham, 1998). Two-fold serial dilutions of PDSTP or antibiotics were prepared into a U-bottom 96-well plate. Bacteria were collected during the mid-log phase of growth and diluted to have about 5 × 10^5^ CFU/mL. In the case of PDSTP alone, the diluted culture was directly inoculated into the 96-well plate, instead, for combinations, the culture was split in two subcultures and PDSTP was added to one of them. Subcultures were inoculated into the 96-well plate which was then incubated at 37°C for 24 h. After the incubation, 30 μL of 0.01% resazurin (Sigma-Aldrich) were added to each well and the plate was further incubated at 37°C for 4 h. Blue to purple resazurin is reduced to pink resorufin by aerobic respiration of metabolically active bacterial cells, allowing visual determination of the MICs. Results are also confirmed by quantifying resorufin fluorescence (ex: 520 nm, em: 580–640 nm) using a GloMax Discover (Promega) microplate reader.

### 2.5 Time-killing assays

#### 2.5.1 Time-killing assay by plate counting

Time-killing assays were performed in TSB to evaluate the activity of PDSTP in combination with different antibiotics against *P. aeruginosa* PA01 overtime, using the broth microdilution method (Scoffone et al., 2015). An overnight liquid bacterial culture was diluted 1: 100 and incubated as a shaking culture (200 rpm) at 37°C. Bacterial growth was followed by monitoring the OD_600_ and, once the culture reached an optical density of 0.35 (corresponding to about 1 × 10^8^ CFU/mL), it was divided into four subcultures. One subculture was not treated (control), the second one was supplemented with sub-inhibitory concentrations of PDSTP, the antibiotic was added to the third subculture at a concentration equivalent to a half of the MIC, and the last one was treated with both PDSTP and the antibiotic. Subcultures were further incubated under the same conditions. At each time point (0, 1, 2, 3, 4, 5 and 24 h), the OD_600_ was measured and bacterial viable count was evaluated by plating appropriate dilutions of the collected aliquots to calculate the respective number of CFU/mL. Time-killing curves were generated by plotting the ∆log_10_ (CFU/mL) versus time, where the ∆log_10_ (CFU/mL) represents the difference in log_10_ (CFU/mL) of each time point compared to the log_10_ (CFU/mL) at time 0.

Synergy was defined as a ≥ 2 log_10_ decrease in the CFU/mL of the culture treated with the antibiotic-PDSTP combination after 5 h compared with its most active component (CLSI, 1999).

#### 2.5.2 High-throughput time-killing assay using the plate reader

The time-killing assay experimental procedure was modified, obtaining a high-throughput protocol to test the activity of PDSTP combinations with antibiotics against the *CF* clinical isolates at once. Overnight bacterial cultures were diluted 1:100 in TSB and incubated as shaking cultures (200 rpm) at 37°C, until an OD_600_ of 0.35 was reached. 200 μL/well of bacterial cultures were transferred into a U-bottom 96-well plate and supplemented with sub-inhibitory concentrations of PDSTP, antibiotic or their combination, in duplicate. One well was left untreated as growth control. Assays were carried out using a CLARIOstar microplate reader (BMG Labtech), measuring the OD_600_ of the cultures every 15 min for 24 h at 37°C. To perform these experiments, a custom plate mode program was set up, including 90 cycles with shaking for 900 s (orbital shaking at 300 rpm, with 3 mm of diameter) before each reading, to increase the oxygenation and maintain bacteria in suspension.

### 2.6 Analysis of the PDSTP-antibiotic combination *in vivo*

Combination therapy of PDSTP and ceftazidime was tested *in vivo* by *G. mellonella* infection experiments (Benthall et al., 2015). Larvae were purchased from a local provider in Pavia and grouped in petri dishes (at least 10 larvae/group) according to their weight. Subsequently, inoculation with a lethal dose (10^4^ CFU) of mid-exponential phase *P. aeruginosa* PA01, or physiological saline (control), was carried out with an injection volume of 10 μL. After 2 h of incubation at 30°C in the dark, mock and PA01 infected larvae were administered with 10 μL of physiological saline (control), ceftazidime (5 mg/kg), PDSTP (6.25 mg/kg) or a combination of ceftazidime (5 mg/kg) and PDSTP (6.25 mg/kg), and re-incubated in the same conditions for 3 days. Larval viability was registered after 24, 48 and 72 h, considering the lack of movement after tactile stimulus, suggestive of larval death.

### 2.7 *In vitro* biofilm inhibition test in 96-well microtiter plates and by confocal laser scanning microscopy

The biofilm inhibitory activity of PDSTP was tested on *P. aeruginosa* strains using the crystal violet staining method (Vandecandelaere et al., 2016). An overnight bacterial culture in TSB was diluted to an OD_600_ of about 0.05 (corresponding to approximately 1 × 10^8^ CFU/mL) in fresh medium. 200 μL of the culture were inoculated into the microtiter plate and incubated for 2 h at 37°C. After the incubation, the supernatant was removed and replaced with 200 μL of fresh medium with or without 50 μg/mL of PDSTP. The plate was further incubated for 20 h at 37°C. Biofilm biomass was then quantified by crystal violet staining. For the confocal laser scanning microscopy biofilm visualization, an overnight bacterial culture in TSB was diluted to an OD_600_ of about 0.05 in fresh medium. The bacterial suspension was inoculated into the four-well chambered coverslip μ-Slide Glass Bottom (Ibidi) for 2 h at 37°C. After the incubation, the supernatant was removed and replaced with fresh TSB medium with or without 25–50 μg/mL of PDSTP. After overnight incubation, the medium was removed, biofilms were washed twice with PBS and stained with Syto 9 (Invitrogen) at a final concentration of 1 μM. Samples were visualized with a Leica TCS SP8 confocal microscope equipped with a 63× oil immersion objective (HC Pl Apo CS2 63x oil/1.4, Leica). A 488 nm laser line was used to excite Syto9 fluorescence and the emission was collected between 500 and 550 nm. Three snapshots were acquired randomly at different positions in the confocal field of each chamber. The Z-slices were obtained every 0.3 microns. For visualization and processing of biofilm images, ImageJ was used. Thickness, biomass, roughness coefficient and biofilm distribution were analyzed using the COMSTAT2 software (Heydorn et al., 2000).

### 2.8 *Ex vivo* pig lung biofilm model

#### 2.8.1 *Ex vivo* pig lung dissection and infection

All pig lungs used were supplied by a commercial butcher and dissected on the day of arrival. *Ex vivo* pig lung (EVPL) tissue was dissected to extract the bronchioles as previously described (Harrington et al., 2021). Following UV light sterilization, square bronchiolar tissue pieces were placed into a 24-well plate with a 400 μL, 0.8% agarose pad (UV sterilized).

To infect the tissue, 2 μL of an overnight *P. aeruginosa* culture diluted to an OD_600_ of about 0.05 were spotted onto the tissue, while 2 μL of SCFM were spotted as negative control. To better mimic the *CF* lung environment, 500 μL SCFM were added to each well and the plate was incubated statically for the 2 h at 37°C. After the incubation, medium was removed and replaced with 500 μL of fresh SCFM with or without 25–50 μg/mL of PDSTP. 0.06 μg/mL of ciprofloxacin were used as positive control. The plate was incubated overnight at 37°C.

#### 2.8.2 Bacterial recovery from the EVPL model and count determination

To recover bacteria, tissues were removed from the wells and washed with 500 μL of PBS. Tissue sections were then transferred in sterile homogenization tubes (Fisherbrand) with 5 mm glass beads (Merck) and 500 μL of PBS. Samples were homogenized using a Minilys Homogenizer (Bertin) for 20 s at 15 m/s. Tissue homogenates were serially diluted in PBS and plated on LB agar. Colony counts were performed after 24 h at 37°C.

#### 2.8.3 Biofilm staining on the EVPL model

After overnight incubation, tissues were washed with 500 μL of PBS and placed into four-well chambered coverslip μ-Slide Glass Bottom (Ibidi). Tissues were stained with 5 μM Syto9 (Invitrogen), washed twice with PBS and turned upside down onto the cover glass. Samples were visualized as described for *in vitro* biofilm analysis. The scaffold surface was acquired using a 561 nm laser in reflection mode. Three Z-stacks randomly chosen at different positions in each chamber were acquired with a *Z* step of 0.7 microns.

### 2.9 Graphical representations and statistical analyses

GraphPad Prism version 10.0 was utilized to create visual representations and perform statistical analyses. The graphical representations illustrate average values along with their respective standard deviations. Group variances were evaluated using Student’s *t* test, one-way ANOVA or the Log-rank (Mantel-Cox) test. Significance was determined at a threshold of *p* < 0.05.

## 3 Results

### 3.1 PDSTP impairs *Pseudomonas aeruginosa* adhesion to human pulmonary epithelial cells

Prior to investigate the putative anti-adhesive properties of PDSTP, its antibacterial activity was tested by broth microdilution method in MHB and TSB against the reference strain *P. aeruginosa* PA01 and a panel of 9 *P. aeruginosa CF* clinical isolates (Alcalá-Franco et al., 2012). As reported in Table 1, the compound showed a negligible effect on *P. aeruginosa* growth.

**Table 1:** Minimum inhibitory concentrations of PDSTP for *Pseudomonas aeruginosa* PA01 and a panel of 9 *Pseudomonas aeruginosa* CF clinical isolates in MHB and TSB.

		PA01	BST44	SG2	NN2	NN83	NN84	RP73	RP74	BT2	BT72
MIC (μg/mL)	**MHB**	64	256	128	64	32	64	512	512	32	32
	**TSB**	256	512	256	256	128	256	>512	512	256	128

**Figure 2:**
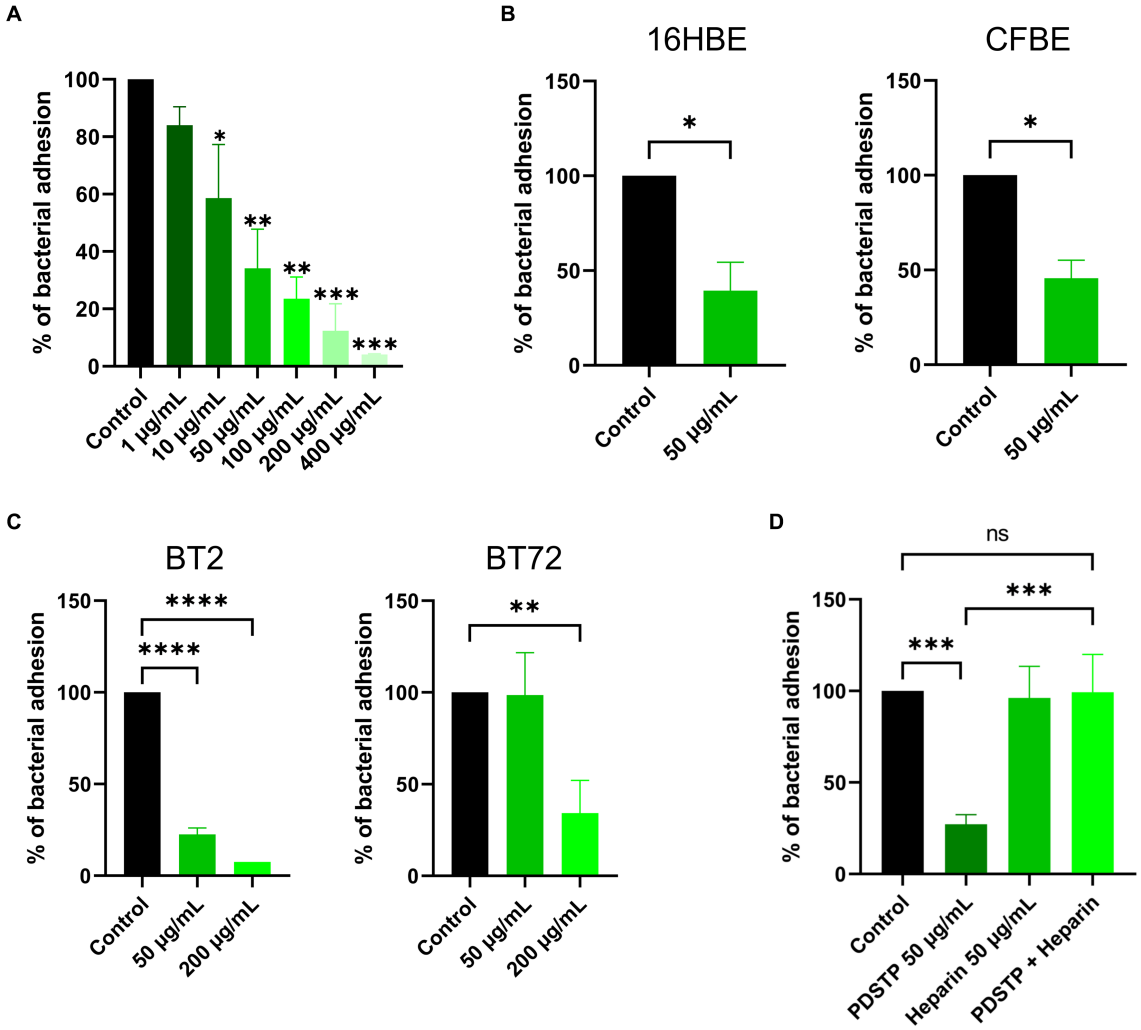
Differential adhesion abilities to immortalized human cell lines of *Pseudomonas aeruginosa* strains treated with PDSTP. Adhesion of *Pseudomonas aeruginosa* PA01 on A549 cell monolayers in the presence of increasing concentrations of PDSTP (from 1 to 400 μg/mL) **(A)**. Adhesion of *Pseudomonas aeruginosa* PA01 on 16HBE14o- and CFBE41o- cell monolayers in the presence of 50 μg/mL of PDSTP **(B)**. Adhesion of *Pseudomonas aeruginosa* BT2 and BT72 on A549 cell monolayers in the presence of 50 and 200 μg/mL of PDSTP **(C)**. Adhesion of *Pseudomonas aeruginosa* PA01 on A549 cell monolayers in the presence of 50 μg/mL of PDSTP, 50 μg/mL heparin and a mixture of PDSTP and heparin (at the previously used concentrations) **(D)**. Each experiment was performed three times, each time in duplicate. Statistically significant differences are indicated (One-way ANOVA test for panel **(A,C,D)**; ns, not significant; *, *p* < 0.05; **, *p* < 0.01; ***, *p* < 0.001; ****, *p* < 0.0001. Student’s *t* test for panel **(B)**; *, *p* < 0.05).

Then, the anti-adhesive effect of PDSTP was evaluated through adhesion assays. Initially, PDSTP concentrations close to or above the MIC against *P. aeruginosa* PA01 (64 μg/mL in DMEM and 128 μg/mL in MEM) were evaluated to inhibit the adhesion to epithelial cells at 37°C. In these conditions, bacterial growth was partially affected, resulting in confounding results. Consequently, adhesion assays were performed at 4°C (Berlutti et al., 2008), where PDSTP, even at high concentrations, did not impair bacterial viability, while human cells viability (visually evaluated at the microscope) was not affected (data consistent with Schmidtke et al., 2002). *P. aeruginosa* PA01 adhesion to the A549 lung epithelial cell line was impaired in a dose-dependent manner when treated with increasing concentrations of PDSTP (from 1 to 400 μg/mL). A statistically significant decrease in bacterial adhesion was already appreciated by treating cells with 10 μg/mL of PDSTP (41.4% reduction compared to the control), while it was almost completely abolished with a treatment of 400 μg/mL (95.9% reduction compared to the control) (Figure 2A).

PDSTP (50 μg/mL) also affected the adhesion of *P. aeruginosa* PA01 to the 16HBE14o- (WT-CFTR) and CFBE41o- (ΔF508-CFTR) bronchial cell lines, reducing bacterial adhesion of 60.6 and 54.3%, respectively (Figure 2B).

The anti-adhesive properties of PDSTP were then tested against hypermucoid *P. aeruginosa* strains. The activity of PDSTP was analysed on a *P. aeruginosa* clone, specifically BT2 (early isolate) and BT72 (late isolate) strains, isolated from the same *CF* patient 15 years apart. Interestingly, *P. aeruginosa* BT2 adhered more to A549 cells compared to the BT72 strain (data not shown), a phenotype that has previously been described in longitudinal studies (Hawdon et al., 2010). In this experiment, two concentrations of PDSTP were assayed. As shown in Figure 2C, *P. aeruginosa* BT2 adhesion was 22.5 and 7.5% that of the control when treated with 50 and 200 μg/mL of the compound, respectively. On the other hand, 50 μg/mL of PDSTP were not able to affect *P. aeruginosa* BT72 adhesion, but increasing the compound to 200 μg/mL caused a reduction of 65.8% in bacterial adhesion compared to the control (Figure 2C).

Heparin is a structural analogue of heparan-sulphates (Liu and Thorp, 2002). Since PDSTP binds to heparan-sulphates (Schmidtke et al., 2003), an excess of heparin should scavenge the compound, allowing bacteria to interact with HSGAGs and consequently to adhere to human cells. To test this hypothesis, A549 cell monolayers were treated with PDSTP (50 μg/mL), heparin (50 μg/mL) and an equal combination of these two compounds (50 μg/mL each). As a result, heparin alone did not impair *P. aeruginosa* PA01 adhesion to human cells, while the combination of PDSTP and heparin re-established the normal adhesive properties of the bacterium to the A549 cell line (Figure 2D).

**Figure 3:**
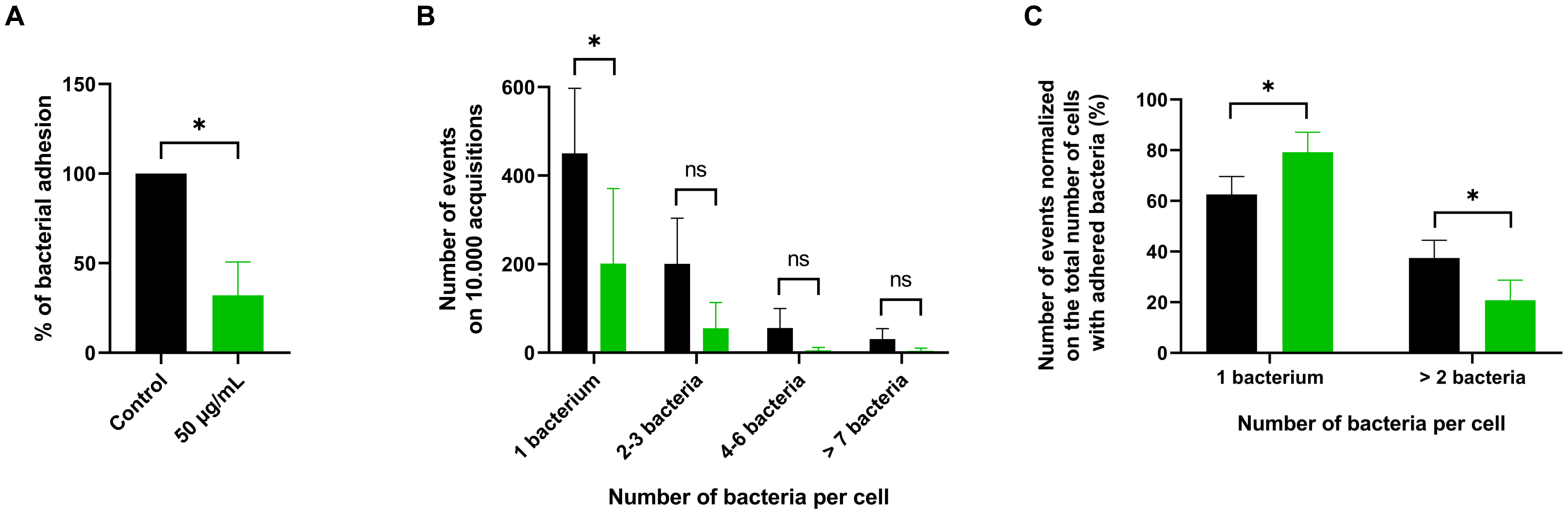
ImageStream flow cytometry analysis of PDSTP anti-adhesive effect against *Pseudomonas aeruginosa* PA01. Adhesion of GFP-expressing *Pseudomonas aeruginosa* PA01 on A549 cell monolayers in the presence of 50 μg/mL of PDSTP **(A)**. Events showing the number of adhered GFP-expressing *Pseudomonas aeruginosa* PA01 per A549 cell on 10.000 acquisitions of the control (black column) and samples treated with 50 μg/mL of PDSTP (green column) **(B)**. Number of events normalized on the total number of cells with adhered bacteria, expressed in percentage, of the control (black column) and cells treated with 50 μg/mL of the compound (green column) **(C)**. The experiment was performed twice, each time in duplicate. Statistically significant differences are indicated (Student’s *t* test; ns, not significant; *, *p* < 0.05).

To validate these results, adhesion was quantified by imaging flow cytometry. GFP-expressing *P. aeruginosa* PA01 adhesion to the A549 cell line showed a reduction of 68% when treated with 50 μg/mL of PDSTP, compared to the control (Figure 3A). This is consistent with the adhesion reduction reported in Figure 2A using the same concentration of the compound (66% reduction).

Imaging flow cytometry can also provide a software-based analysis of the fluorescent spots within single-cell images (Supplementary Figure S1), allowing a more detailed characterization of the PDSTP anti-adhesive effect. The number of adhered GFP-expressing *P. aeruginosa* PA01 bacteria for each A549 cell was counted and data were represented in terms of number of events, i.e., the number of human cells with 1, 2–3, 4–6 or more than 7 adhered bacteria, on 10.000 acquisition (Supplementary Figure S2). As shown in Figure 3B, most of the control human cells interacted with only one bacterium, while the number of events involving a larger number of bacteria decreased proportionally. The same pattern was observed in samples treated with 50 μg/mL of PDSTP, but with fewer events due to impaired bacterial adhesion. Interestingly, when the numbers of events were normalized on the total number of cells with adhered bacteria, it was evident that PDSTP especially affected multiple bacterial adhesion (Figure 3C).

### 3.2 PDSTP potentiates the activity of antibiotics currently used in clinics

Combination therapy involves the combination of antibiotics with compounds that increase their intracellular concentration or allow to overcome antibiotic resistance (Wang et al., 2020). To determine whether PDSTP could potentiate the activity of antibiotics currently used to treat *P. aeruginosa* infections, antibiotic combination susceptibility tests were performed. Antibiotics with different mechanisms of action were chosen, specifically amikacin, ceftazidime, ciprofloxacin, colistin, meropenem and tobramycin. This panel of antibiotics was combined with PDSTP and tested against *P. aeruginosa* PA01. Specifically, PDSTP was used at a concentration of 25 μg/mL, which corresponds to about half of the MIC in MHB (Table 1).

As reported in Table 2, the MICs of the tested antibiotics showed a reduction ranging from 2 to 128-fold when combined with PDSTP. In particular, the presence of PDSTP did not cause a great increase in tobramycin efficacy (2-fold decrease in the MIC), while for all the other antimicrobials, especially for ceftazidime (128-fold decrease in the MIC), an adjuvant effect was observed.

**Table 2:** Minimum inhibitory concentrations in MHB of a panel of antibiotics and those of their combinations with PDSTP against *Pseudomonas aeruginosa* PA01 and respective fold reduction.

*Pseudomonas aeruginosa* PA01	Susceptible (S)	Intermediate (I)	Resistant (R)	MIC	MIC antibiotic + PDSTP	Fold reduction
Amikacin	≤ 16 μg/mL	32 μg/mL	≥ 64 μg/mL	4 μg/mL (S)	1 μg/mL (S)	4x
Tobramycin	≤ 4 μg/mL	8 μg/mL	≥ 16 μg/mL	0.5 μg/mL (S)	0.25 μg/mL (S)	2x
Ceftazidime	≤ 8 μg/mL	16 μg/mL	≥ 32 μg/mL	2 μg/mL (S)	0.0156 μg/mL (S)	128x
Meropenem	≤ 2 μg/mL	4 μg/mL	≥ 8 μg/mL	1 μg/mL (S)	0.0625 μg/mL (S)	16x
Ciprofloxacin	≤ 0.5 μg/mL	1 μg/mL	≥ 2 μg/mL	0.125 μg/mL (S)	0.0078 μg/mL (S)	16x
Colistin	-	≤ 2 μg/mL	≥ 4 μg/mL	1 μg/mL (I)	0.0625 μg/mL (I)	16x

MIC breakpoints used to categorize *Pseudomonas aeruginosa* as susceptible, intermediate or resistant to antibiotics are shown. Susceptible (S), intermediate (I) and resistant (R) antibiotic profile are indicated below the respective MIC value.

Subsequently, combination efficacy was tested on multidrug-resistant *P. aeruginosa CF* clinical strains (BST44, SG2, NN2, NN83, NN84, RP73, RP74, BT2, and BT72) only for those antibiotics that showed, according to the Clinical and Laboratory Standard Institute (CLSI) guidelines (CLSI, 2020), an intermediate or resistant profile (Table 3). Also in this case, the concentration of PDSTP in the combination was around half of the MIC determined for each bacterium in MHB (Table 1), i.e., 100 μg/mL for BST44, 50 μg/mL for SG2, 25 μg/mL for NN2,15 μg/mL for NN83, 25 μg/mL for NN84, 200 μg/mL for RP73 and 200 μg/mL for RP74. As indicated in Table 3, there was an overall increased activity when antibiotics were combined with PDSTP. Only for tobramycin and ciprofloxacin, MIC reduction did not exceed 2-fold for each strain, while amikacin MIC decreased 4-fold only against SG2 strain (Table 3). As reported for PA01 strain, the combination with ceftazidime showed the most remarkable effect, leading to a significant reduction in the MICs of all isolates, up to 128-fold (Table 3). Moreover, meropenem confirmed the promising activity of PDSTP-β-lactam combination, being its efficacy significantly increased in each strain tested, up to 32-fold (Table 3). Generally, all the clinical isolates tested showed resistance to at least two antibiotics. Two strains were even resistant to four antimicrobials (NN84 and RP74). In most cases, PDSTP was able to resensitize the clinical isolates showing a resistant antibiotic profile, especially to meropenem (4/4 strains), ceftazidime (6/7 strains) and amikacin (4/6 strains), while one strain was only partially resensitized, i.e., from resistant to intermediate antibiotic profile (Table 3).

**Table 3:** Minimum inhibitory concentrations in MHB of a panel of antibiotics and of their combinations with PDSTP against *Pseudomonas aeruginosa* CF clinical isolates, along with the respective fold reduction.

		BST44	SG2	NN2	NN83	NN84	RP73	RP74
Amikacin	MIC		64 μg/mL (R)	32 μg/mL (I)	256 μg/mL (R)	32 μg/mL (I)	64 μg/mL (R)	32 μg/mL (I)
	MIC antibiotic + PDSTP		16 μg/mL (S)	16 μg/mL (S)	128 μg/mL (R)	16 μg/mL (S)	32 μg/mL (I)	16 μg/mL (S)
	Fold reduction		4x	2x	2x	2x	2x	2x
Tobramycin	MIC		8 μg/mL (I)	>256 μg/mL (R)	>256 μg/mL (R)			
	MIC antibiotic + PDSTP		4 μg/mL (S)	>256 μg/mL (R)	>256 μg/mL (R)			
	Fold reduction		2x					
Ceftazidime	MIC	16 μg/mL (I)	128 μg/mL (R)	16 μg/mL (I)	32 μg/mL (R)	16 μg/mL (I)	32 μg/mL (R)	128 μg/mL (R)
	MIC antibiotic + PDSTP	1 μg/mL (S)	2 μg/mL (S)	0.125 μg/mL (S)	4 μg/mL (S)	0.5 μg/mL (S)	4 μg/mL (S)	32 μg/mL (R)
	Fold reduction	16x	64x	128x	8x	32x	8x	4x
Meropenem	MIC	16 μg/mL (R)				32 μg/mL (R)	16 μg/mL (R)	16 μg/mL (R)
	MIC antibiotic + PDSTP	1 μg/mL (S)				1 μg/mL (S)	1 μg/mL (S)	2 μg/mL (S)
	Fold reduction	16x				32x	16x	8x
Ciprofloxacin	MIC					4 μg/mL (R)		16 μg/mL (R)
	MIC antibiotic + PDSTP					2 μg/mL (R)		8 μg/mL (R)
	Fold reduction					2x		2x

Susceptible (S), intermediate (I) and resistant (R) antibiotic profile are indicated below the respective MIC value.

**Figure 4:**
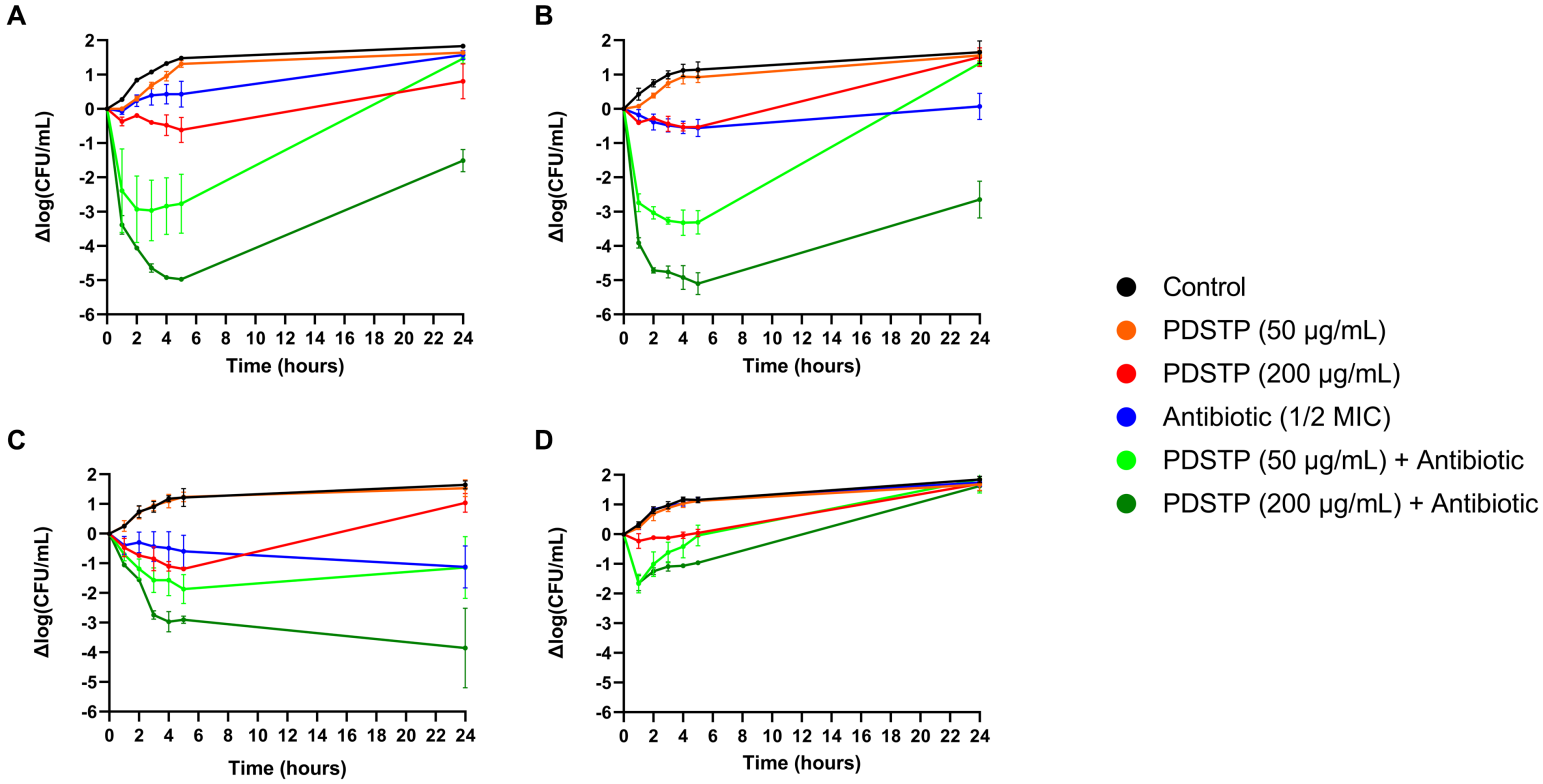
*Pseudomonas aeruginosa* PA01 time-killing assays of tobramycin **(A)**, ciprofloxacin **(B)**, ceftazidime **(C)** and colistin **(D)** combined with either 50 or 200 μg/mL of PDSTP, represented as the difference in log_10_(CFU/mL) of each time point against the log_10_ (CFU/mL) at time 0. Black line, untreated sample; orange line, treatment with 50 μg/mL of PDSTP; red line, treatment with 200 μg/mL of PDSTP; blue line, treatment with a concentration equal to ½ MIC of the antibiotic; light green line, combination of 50 μg/mL of PDSTP with the antibiotic; dark green line, combination of 200 μg/mL of PDSTP with the antimicrobial.

To better characterize the activity of the combinations, *P. aeruginosa* PA01 time-killing assays in TSB were performed. As reported in Figure 4, combination of tobramycin (1 μg/mL), ciprofloxacin (0.0625 μg/mL), ceftazidime (1 μg/mL) and colistin (1 μg/mL), at a concentration equivalent to half of the respective MIC in TSB (data not shown), with either 50 or 200 μg/mL of PDSTP, showed a significant reduction in bacterial viability respect to the best treatment with the only antibiotic or PDSTP, especially during the first hours of treatment. As expected, combinations with 200 μg/mL of PDSTP had a greater activity compared to those with 50 μg/mL. In particular, combination of tobramycin with 50 and 200 μg/mL of PDSTP (Figure 4A) showed a bacterial viability reduction up to 3.48 and 4.45 log_10_ in the CFU/mL. On the other hand, 50 and 200 μg/mL of the PDSTP combined with ciprofloxacin (Figure 4B) reduced bacterial viability up to 2.78 and 4.54 logs, respectively. Since reductions in the CFU/mL after 5 h of treatment were greater than 2 log_10_ compared to the respective most active compound, combinations were defined as synergistic at the lowest tested concentration of PDSTP. On the other hand, combination of PDSTP with either ceftazidime (Figure 4C) and colistin (Figure 4D) did not show synergy (up to 0.72/1.89 log_10_ CFU/mL reduction for ceftazidime and 1.43/1.42 log_10_ CFU/mL reduction for colistin). Interestingly, with the administration of 50 μg/mL of PDSTP in combination with the antibiotics, bacterial viability after 24 h was comparable with the control growth for tobramycin, ciprofloxacin and colistin, and with the antibiotic treatment alone for ceftazidime. In contrast, when antimicrobials were combined with 200 μg/mL of PDSTP, the number of CFU/mL after 24 h was, for each antibiotic but colistin, significantly lower compared to the other conditions, especially for ceftazidime (Figure 4).

To confirm the results obtained with PA01 strain, the efficacy of the same PDSTP-antibiotic combinations was also tested against the 9 *P. aeruginosa CF* clinical isolates by time-killing assays in TSB using a plate reader. In this case, considering the fair correlation between CFU reduction and the OD_600_ variations seen in time-killing experiments with PA01 strain (Supplementary Figure S3), bacterial growth was monitored for 24 h by measuring the OD_600_. The concentrations of PDSTP and antibiotic employed differ for each strain (Supplementary Tables S1–S4) since they were chosen to avoid the complete inhibition of the growth with the monotherapy. With a few exceptions, these concentrations corresponded to about half of the MIC value of ceftazidime, tobramycin and ciprofloxacin, while the concentrations of colistin were equal to the MIC (Supplementary Table S5). In this assay, bacterial growth was considered completely inhibited after the treatment when the increase in OD_600_ did not exceed 0.3.

In general, combination therapies caused a significant inhibition of the bacterial growth, although this was not the case for RP73 and RP74 strains, against which PDSTP was unable to synergize with any of the four antibiotics (Supplementary Tables S1–S4; Supplementary Figure S4–S7) and so they were considered resistant to the PDSTP antibiotic-enhancing activity under these experimental conditions. The most promising results were obtained with the PDSTP-ceftazidime combination. Indeed, this combination induced complete inhibition of bacterial growth for 24 h for each *P. aeruginosa* strain, including the highly resistant SG2 strain (Supplementary Table S1; Supplementary Figure S4). Instead, the combination with tobramycin showed a more variable efficacy, resulting in a 24-h growth inhibition of SG2, NN84, BT2 and BT72 strains, and only a 6-h inhibition for the BST44 strain (Supplementary Table S2; Supplementary Figure S5). Moreover, PDSTP could not revert the tobramycin resistant phenotype of NN2 and NN83 strains (Supplementary Table S2), as already highlighted by antibiotic combination susceptibility testing (Table 3; Supplementary Figure S5). PDSTP-ciprofloxacin combination showed a long-term inhibitory effect against each strain, although a complete growth inhibition was observed only for NN2, NN84, BT2 and BT72 (Supplementary Table S3; Supplementary Figure S6). Finally, as reported for PA01, colistin resulted the least effective antibiotic against *P. aeruginosa*. Indeed, even using a concentration equal to the MIC, its combination with PDSTP led to a long-term inhibition only against NN84 strain, while a 10-h inhibition was observed for BST44, NN2, NN83, BT2 and BT72 (Supplementary Table S4; Supplementary Figure S7). SG2 strain was insensitive to this combination (Supplementary Table S4; Supplementary Figure S7).

Overall, time-killing assays confirmed the results obtained by antibiotic combination susceptibility testing, validating the efficacy of PDSTP as enhancer of the activity of different classes of clinically relevant antibiotics against *P. aeruginosa* in the tested conditions.

### 3.3 PDSTP increases ceftazidime efficacy *in vivo*

**Figure 5:**
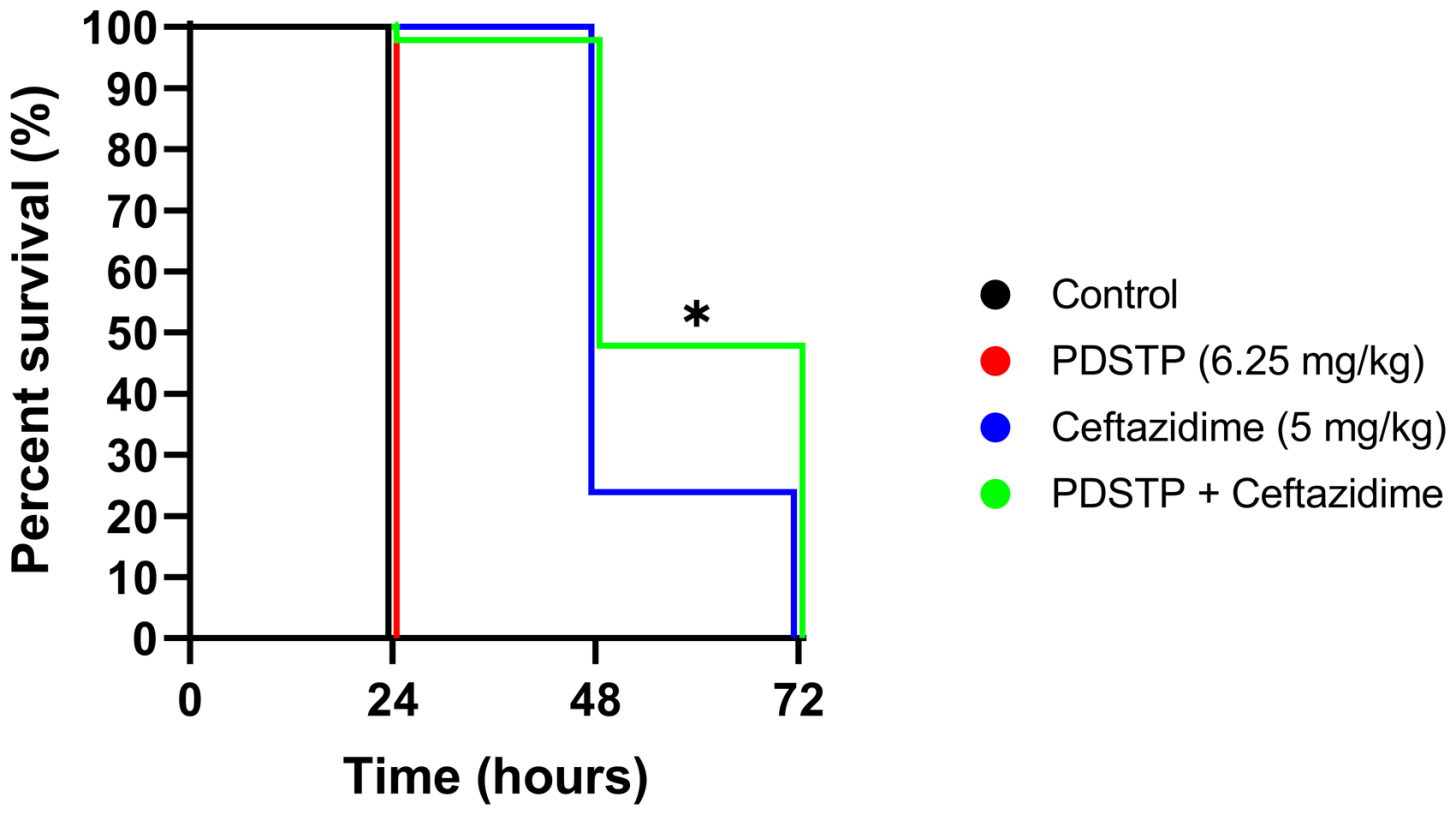
Kaplan–Meier survival curve of *Galleria mellonella* larvae infected with *Pseudomonas aeruginosa* PA01 and treated with physiological saline (black), PDSTP (red), ceftazidime (blue) or the combination of PDSTP and ceftazidime (green). The experiment was performed three times. Statistically significant differences are indicated (Log-rank test; *, *p* < 0.05).

The *in vitro* efficacy of the PDSTP-ceftazidime combination was validated *in vivo* by *Galleria mellonella* infection experiments. Ceftazidime (5 mg/kg) and PDSTP (6.25 mg/kg) concentrations employed in this assay were non-toxic to *G. mellonella* (data not shown). Infection with 10^4^ CFU of *P. aeruginosa* PA01 caused 100% larval mortality in the non-treated (data not shown), physiological saline and PDSTP treated groups (Figure 5) 24 h post-inoculation. On the contrary, treatment with ceftazidime alone led to 25% survival after 48 h. Of note, the combination treatment with PDSTP gave a statistically significant increase in larval viability compared with the antibiotic alone up to 47% at 48 h (Figure 5). This confirms the *in vitro* activity observed for the PDSTP-ceftazidime combination.

### 3.4 PDSTP inhibits biofilm formation *in vitro* and in an *ex vivo* pig lung model

**Figure 6:**
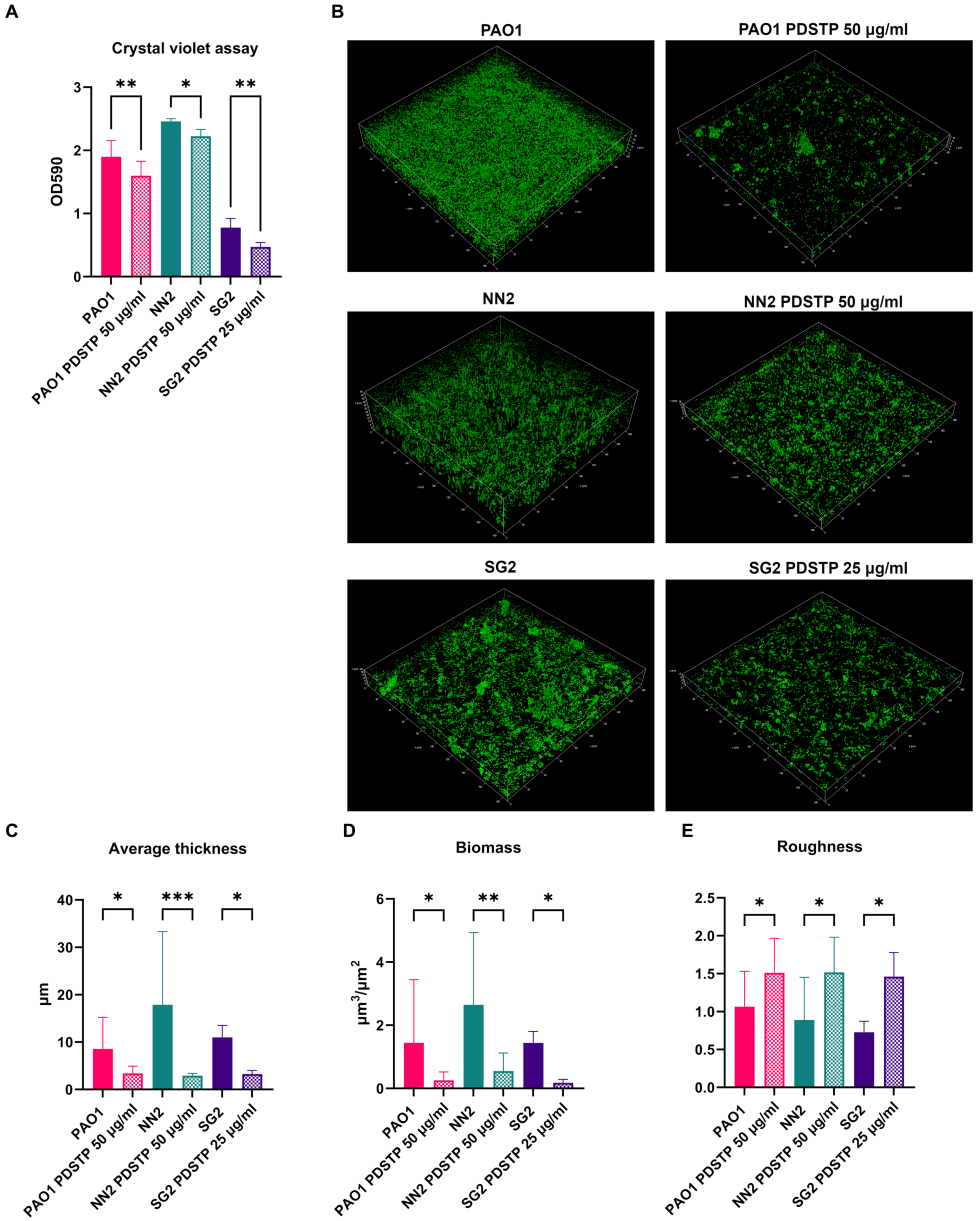
Effect of PDSTP against biofilm formation in *Pseudomonas aeruginosa* PA01, NN2 and SG2 strains using crystal violet assay **(A)**. CLSM images (400x magnification) of *Pseudomonas aeruginosa* biofilms formed with or without PDSTP **(B)**. 2D images acquired at equal distances along the *Z*-axis were stacked to reconstruct the 3D biofilm images. Analysis of biofilm properties by COMSTAT2 **(C–E)**. Experiments were performed three times. Statistically significant differences are indicated (One-way ANOVA test; *, *p* < 0.05; **, *p* < 0.01; ***, *p* < 0.001).

To evaluate whether PDSTP affects biofilm formation of *P. aeruginosa* PA01 and two clinical isolates, *in vitro* biofilm inhibition assays were performed in TSB. PA01, NN2 and SG2 strains were employed due to their differences in biofilm formation abilities. First, crystal violet assays showed that 50 μg/mL of PDSTP (or 25 μg/mL for SG2 since 50 μg/mL inhibited the growth in the experimental conditions described in Materials and Methods) significantly decreased the quantity of biofilm formed after 24 h compared to the respective untreated controls (Figure 6A).

PDSTP antibiofilm activity was then evaluated using confocal laser scanning microscopy (CLSM). In particular, PA01 strain formed a thick and homogeneous biofilm. Compared to PA01, NN2 biofilm was thicker, while that of SG2 was more heterogeneous and characterized by bacterial aggregates (Figure 6B). For all the strains, PDSTP impaired biofilm formation, i.e., the quantity of biofilm was visibly decreased and SG2 biofilm was less structured (Figure 6B). Subsequent COMSTAT2 analysis showed that the compound significantly reduced both the average biofilm thickness and biomass (Figures 6C,D), while it significantly increases the roughness, an indicator of altered biofilm structure (Figure 6E).

**Figure 7:**
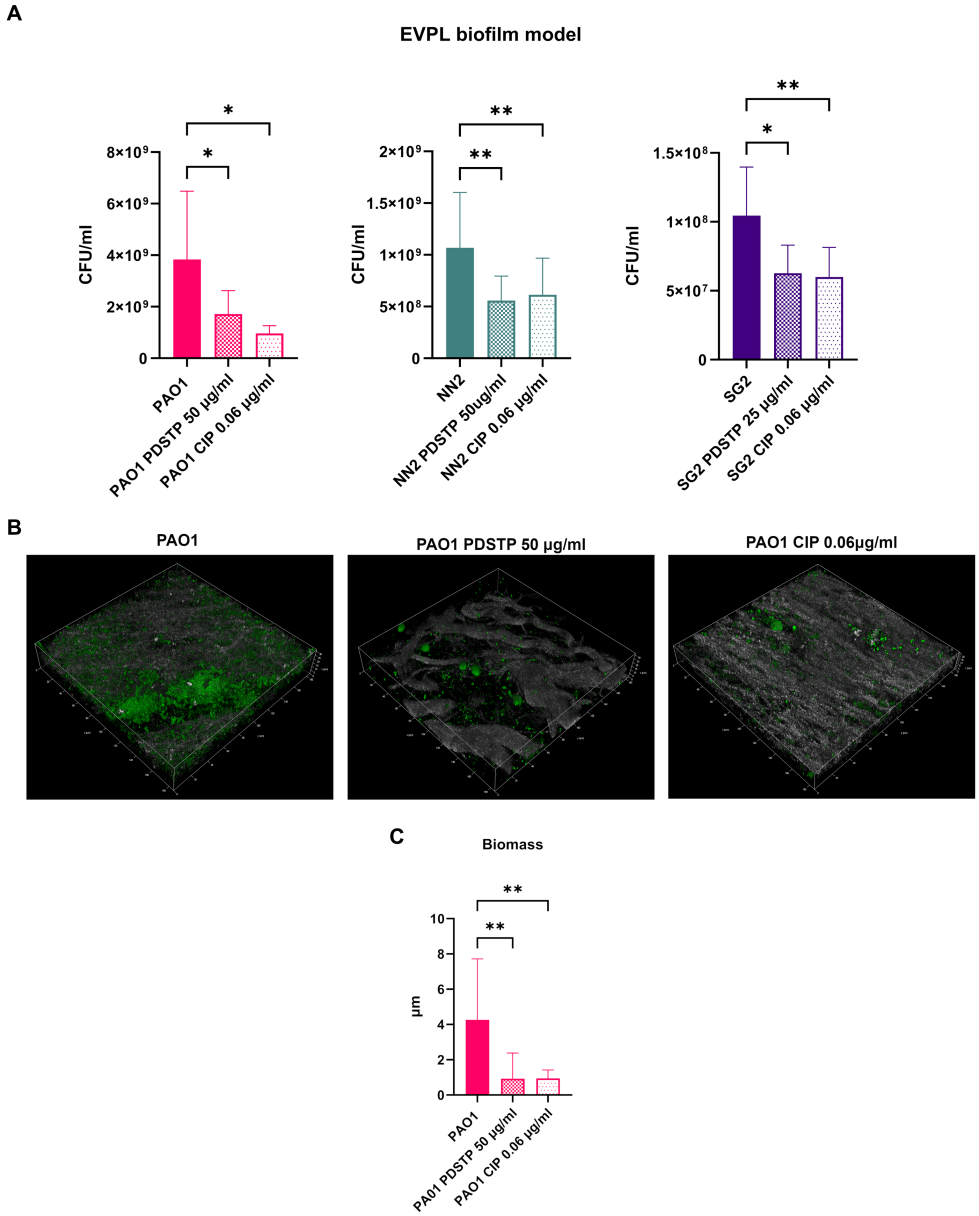
Effect of PDSTP on *Pseudomonas aeruginosa* PA01, NN2 and SG2 strains biofilm formation in an *ex vivo* pig lung model (EVPL), represented as the number of CFU/mL recovered from treated and untreated tissues **(A)**. CLSM images (400x magnification) of *Pseudomonas aeruginosa* PA01 biofilms formed on EVPL with or without PDSTP **(B)**. 2D images acquired at equal distances along the *Z*-axis were stacked to reconstruct the 3D biofilm images. Analysis of biofilm properties by COMSTAT2 **(C)**. Experiments were performed three times. Statistically significant differences are indicated (One-way ANOVA test; *, *p* < 0.05; **, *p* < 0.01).

To validate PDSTP antibiofilm activity, an *ex vivo* pig lung (EVPL) tissue model, embedded in synthetic cystic fibrosis medium (SCFM) (Palmer et al., 2007) to further mimic the CF lung environment, was employed. The pig lung structure and immunology resemble those of humans, making this model particularly relevant. Similarly to the previous analyses, biofilm formation was impaired by the compound for all the strains. Indeed, the number of CFU/mL recovered from biofilms were significantly lower when treated with PDSTP, compared to untreated controls. CFU/mL reduction was similar to that of ciprofloxacin treatment (0.06 μg/mL), used as positive control (Figure 7A). Finally, CLSM was used to visualize *P. aeruginosa* PA01 biofilm, stained with Syto9, on lung tissue fragments. Also in this case, PDSTP (50 μg/mL) impaired biofilm formation (Figure 7B), while COMSTAT2 analysis showed the significant reduction in biofilm biomass due to PDSTP treatment, compared to the control, which is in line with ciprofloxacin (0.06 μg/mL) treatment (Figure 7C).

## 4 Discussion

The antiviral dispirotripiperazine-based compound PDSTP was tested *in vitro* against *P. aeruginosa* PA01 adhesion on different pulmonary epithelial cell lines, including a *CF* cell line, and was effective at very low concentrations. Noteworthy, these concentrations are more than 50 times lower than the 50% cytotoxic concentration (Schmidtke et al., 2002). PDSTP anti-adhesive activity was maintained also when tested against hypermucoid *P. aeruginosa* BT2 and BT72 clinical isolates, although a higher concentration was required to impair the adhesion of BT72. This difference may be attributed to their distinct levels of interaction with human cells.

A preliminary study of the mechanism involved in PDSTP impairment of bacterial adhesion was carried out analysing PA01 strain adhesion by imaging flow cytometry. Interestingly, PDSTP treatment particularly affected the adhesion of multiple bacteria to human cells, probably reducing the accessibility of the human cell surface receptors to bacteria. Indeed, the presence of heparin in adhesion experiments re-established the normal adhesive capabilities of PA01 strain by scavenging PDSTP. This result highlights the high affinity of the compound towards HSGAGs and a possible involvement of these surface receptors in PDSTP anti-adhesive activity as in the case of viral adsorption. Further studies are ongoing in our laboratory to clarify this molecular mechanism.

Considering the current research on anti-adhesive molecules against *P. aeruginosa*, PDSTP shows some advantages compared to other approaches such as natural extracts (Ahmed et al., 2014; Molina Bertrán et al., 2022) and glycoclusters (Malinovská et al., 2019). Indeed, PDSTP has been extensively tested *in vivo* as antiviral, showing to be non-cytotoxic, effective at low concentrations and characterized by a broad therapeutic index. On the contrary, natural extracts and glycoclusters generally require very high concentrations to be effective *in vitro*, often in the range of mg/mL, which limits their use *in vivo*. Moreover, by targeting HSGAGs on cell surface, PDSTP shows a novel anti-adhesive mechanism, different from the inhibition of the lectin-glycan interaction exerted by glycoclusters (Wojtczak and Byrne, 2022). It is worth noting that the compound may have broad-spectrum anti-adhesive properties since other relevant pathogens adhere to HSGAGs, including *Mycobacterium tuberculosis*, Gram-positive streptococci and species belonging to ESKAPE group (García et al., 2016).

By determining the MIC against PA01 and different *CF* strains, the compound showed a low inherent antimicrobial activity with strain-specific susceptibilities. These differences are probably due to modifications of the molecular target which is likely localized on the bacterial surface. This hypothesis is supported by the high molecular weight of the compound, which prevents its diffusion via porins, as well as its high polarity, which makes its entry across the lipid bilayer unlikely (O'Shea and Moser, 2008). A differential effect of PDSTP was also found when MICs were determined using different microbial culture media, with a systematic increase in the MIC values in TSB compared to MHB. A similar culture medium-dependent activity was already reported for aminoglycosides which showed a decreased efficacy in media with high ionic strength, such as TSB. In fact, salt interferes with the electrostatic interactions between aminoglycosides and components of the outer membrane surface that mediate antibiotic uptake (Hancock, 1981). Being the positively charged nitrogen atoms of PDSTP the mediators of the antiviral activity of dispirotripiperazines (Egorova et al., 2021), it is plausible to hypothesize that these charges may also play a role in this specific biological activity.

The slight inhibitory effect on bacterial growth showed by PDSTP is a peculiar characteristic of weak membrane-perturbing antibiotic adjuvants (Douafer et al., 2019). The PDSTP adjuvant activity was proven against the *P. aeruginosa* PA01 and a panel of multidrug-resistant *CF* isolates, showing an overall increase in the efficacy of the tested antibiotics. Specifically, β-lactams showed the highest MIC fold reduction and a durable efficacy of the combination over time for most strains. Aminoglycosides and ciprofloxacin, instead, had a great efficacy in combination with PDSTP, particularly during the first hours of treatment, but with a narrower spectrum of activity. Finally, PDSTP-colistin combination had the least durable inhibitory effect on bacterial growth. The PDSTP efficacy profile as antibiotic adjuvant against *P. aeruginosa* can be compared with that of natural polyamines. Indeed, these molecules significantly decreased the MIC of many β-lactams and other low molecular weight antibiotics against *P. aeruginosa*, although only at high concentrations (Kwon and Lu, 2006). The mechanism of action of these molecules was characterized in optimized spermine derivatives, demonstrating to be mediated by inhibition of efflux pumps and increased permeability of the outer membrane (Cadelis et al., 2021; Wang et al., 2022). Given the similarities in the spectrum of activity of spermine and PDSTP, besides being both characterized by reactive positively charged nitrogens (Li et al., 2019; Egorova et al., 2021), it is plausible to speculate that PDSTP may share a similar mechanism of action against *P. aeruginosa*. We are currently performing experiments to verify this hypothesis. In addition to the promising results obtained, the importance of developing PDSTP as adjuvant compound is also underlined by its uncommon efficacy against this extremely drug-resistant pathogen. In fact, many adjuvants show a limited synergy with the antibiotics against *P. aeruginosa* (Vaara et al., 2010; Stokes et al., 2017; Nikolaev et al., 2020; Zhou et al., 2022).

To validate the *in vitro* results, antibiotic potentiation was evaluated using a *Galleria mellonella* infection model, showing that PDSTP enhanced ceftazidime activity and highlighting its translational potential *in vivo*. To establish the most appropriate administration protocol in mammals, further investigations with murine infection models are necessary, allowing to test multiple administration of the adjuvant, an option not available in *G. mellonella*.

The ability to form biofilm is a major virulence factor in *P. aeruginosa* and new molecules able to impair its formation are highly desirable, thus PDSTP biofilm inhibitory potential was assessed. Using different *in vitro* models, PDSTP was demonstrated to significantly decrease biofilm formation at sub-inhibitory concentrations. Confocal microscopy analysis allowed the visualization of the biofilm perturbation in the presence of the compound, highlighting a substantial decrease in the average biofilm thickness and biomass, besides an overall alteration of the structure. Furthermore, PDSTP efficacy was validated in an *ex vivo* pig lung biofilm model, essentially confirming its efficacy as biofilm inhibitor and showing an activity comparable with the antibiotic ciprofloxacin. The coherence in the results obtained against strains showing macroscopic structural differences in their biofilms suggests that PDSTP impairs biofilm formation by targeting an essential mechanism shared by different *P. aeruginosa* strains. In particular, since the compound is added only after the initial adhesion to the surface, it probably affects bacterial aggregation. Indeed, PDSTP could interact with bacterial surface, as hypothesized for its bacterial growth inhibitory activity, and disrupt the cell–cell interaction fundamental for this process. This putative mechanism of action is unique among the currently reported biofilm inhibitors that mainly act as quorum-sensing inhibitors (O'Loughlin et al., 2013; D'Angelo et al., 2018; Wang et al., 2022), competitors of the lectin binding (Bergmann et al., 2016) or repressors of exopolysaccharide production (van Tilburg Bernardes et al., 2017).

To conclude, PDSTP showed a remarkable spectrum of activities against *P. aeruginosa*, being effective in inhibiting bacterial adhesion to epithelial cells, increasing antibiotic efficacy and impairing biofilm formation. In light of these results, the combined antivirulence and antibiotic potentiation properties of PDSTP may help addressing the emerging threat of multidrug-resistant bacterial infections.

## Data availability statement

The original contributions presented in the study are included in the article/Supplementary material, further inquiries can be directed to the corresponding author/s.

## Author contributions

AB: Formal analysis, Investigation, Methodology, Validation, Writing – original draft, Writing – review & editing. GT: Conceptualization, Formal analysis, Investigation, Methodology, Validation, Writing – original draft, Writing – review & editing. VS: Formal analysis, Investigation, Methodology, Writing – review & editing. SI: Formal analysis, Investigation, Methodology, Writing – review & editing. GB: Formal analysis, Validation, Writing – review & editing. OR: Investigation, Methodology, Writing – review & editing. NM: Investigation, Methodology, Writing – review & editing. VM: Resources, Supervision, Writing – review & editing. SB: Conceptualization, Resources, Supervision, Writing – review & editing.

## References

[ref1] Ahmed G. F. Elkhatib W. F. Noreddin A. M. (2014). Inhibition of Pseudomonas aeruginosa PAO1 adhesion to and invasion of A549 lung epithelial cells by natural extracts. J. Infect. Public Health 7, 436–444. doi: 10.1016/j.jiph.2014.01.009, PMID: 24894307

[ref2] Alcalá-Franco B. Montanari S. Cigana C. Bertoni G. Oliver A. Bragonzi A. (2012). Antibiotic pressure compensates the biological cost associated with Pseudomonas aeruginosa hypermutable phenotypes in vitro and in a murine model of chronic airways infection. J. Antimicrob. Chemother. 67, 962–969. doi: 10.1093/jac/dkr587 22294647

[ref3] Alimbarova L. Egorova A. Riabova O. Monakhova N. Makarov V. (2022). A proof-of-concept study for the efficacy of dispirotripiperazine PDSTP in a rabbit model of herpes simplex epithelial keratitis. Antivir. Res. 202:105327. doi: 10.1016/j.antiviral.2022.105327, PMID: 35487465

[ref4] Antimicrobial Resistance Collaborators (2022). Global burden of bacterial antimicrobial resistance in 2019: a systematic analysis. Lancet 399, 629–655. doi: 10.1016/S0140-6736(21)02724-0, PMID: 35065702 PMC8841637

[ref6] Benthall G. Touzel R. E. Hind C. K. Titball R. W. Sutton J. M. Thomas R. J.. (2015). Evaluation of antibiotic efficacy against infections caused by planktonic or biofilm cultures of Pseudomonas aeruginosa and Klebsiella pneumoniae in galleria mellonella. Int. J. Antimicrob. Agents 46, 538–545. doi: 10.1016/j.ijantimicag.2015.07.014 26364845

[ref7] Bergmann M. Michaud G. Visini R. Jin X. Gillon E. Stocker A.. (2016). Multivalency effects on Pseudomonas aeruginosa biofilm inhibition and dispersal by glycopeptide dendrimers targeting lectin Lec a. Org. Biomol. Chem. 14, 138–148. doi: 10.1039/c5ob01682g, PMID: 26416170

[ref8] Berlutti F. Superti F. Nicoletti M. Morea C. Frioni A. Ammendolia M. G.. (2008). Bovine lactoferrin inhibits the efficiency of invasion of respiratory A549 cells of different iron-regulated morphological forms of Pseudomonas aeruginosa and Burkholderia cenocepacia. Int. J. Immunopathol. Pharmacol. 21, 51–59. doi: 10.1177/039463200802100107, PMID: 18336731

[ref9] Bruscia E. Sangiuolo F. Sinibaldi P. Goncz K. K. Novelli G. Gruenert D. C. (2002). Isolation of CF cell lines corrected at Delta F508-CFTR locus by SFHR-mediated targeting. Gene Ther. 9, 683–685. doi: 10.1038/sj.gt.3301741, PMID: 12032687

[ref10] Bucior I. Pielage J. F. Engel J. N. (2012). Pseudomonas aeruginosa pili and flagella mediate distinct binding and signaling events at the apical and basolateral surface of airway epithelium. PLoS Pathog. 8:e1002616. doi: 10.1371/journal.ppat.1002616, PMID: 22496644 PMC3320588

[ref11] Cadelis M. M. Li S. A. Bourguet-Kondracki M. L. Blanchet M. Douafer H. Brunel J. M.. (2021). Spermine derivatives of indole-3-carboxylic acid, indole-3-acetic acid and indole-3-acrylic acid as gram-negative antibiotic adjuvants. Chem. Med. Chem. 16, 513–523. doi: 10.1002/cmdc.202000359, PMID: 33090655

[ref12] CLSI (1999). Methods for determining bactericidal activity of antimicrobial agents; approved guideline. Wayne, PA: Clinical and Laboratory Standards Institute

[ref13] CLSI (Ed.) (2020). “Performance standards for antimicrobial susceptibility testing” in 30th ed. CLSI supplement M100 (Wayne, PA: Clinical and Laboratory Standards Institute)

[ref14] Cozens A. L. Yezzi M. J. Kunzelmann K. Ohrui T. Chin L. Eng K.. (1994). CFTR expression and chloride secretion in polarized immortal human bronchial epithelial cells. Am. J. Respir. Cell Mol. Biol. 10, 38–47. doi: 10.1165/ajrcmb.10.1.7507342 7507342

[ref15] Crabbé A. Liu Y. Matthijs N. Rigole P. De La Fuente-Nùñez C. Davis R.. (2017). Antimicrobial efficacy against Pseudomonas aeruginosa biofilm formation in a three-dimensional lung epithelial model and the influence of fetal bovine serum. Sci. Rep. 7:43321. doi: 10.1038/srep43321, PMID: 28256611 PMC5335707

[ref16] D'Angelo F. Baldelli V. Halliday N. Pantalone P. Polticelli F. Fiscarelli E.. (2018). Identification of FDA-approved drugs as antivirulence agents targeting the pqs quorum-sensing system of Pseudomonas aeruginosa. Antimicrob. Agents Chemother. 62, e01296–e01218. doi: 10.1128/AAC.01296-18, PMID: 30201815 PMC6201120

[ref17] de Kraker M. E. Stewardson A. J. Harbarth S. (2016). Will 10 million people die a year due to antimicrobial resistance by 2050? PLoS Med. 13:e1002184. doi: 10.1371/journal.pmed.1002184, PMID: 27898664 PMC5127510

[ref18] Douafer H. Andrieu V. Phanstiel O. 4th Brunel J. M. (2019). Antibiotic adjuvants: make antibiotics great again! J. Med. Chem. 62, 8665–8681. doi: 10.1021/acs.jmedchem.8b01781 31063379

[ref19] Egorova A. Bogner E. Novoselova E. Zorn K. M. Ekins S. Makarov V. (2021). Dispirotripiperazine-core compounds, their biological activity with a focus on broad antiviral property, and perspectives in drug design (mini-review). Eur. J. Med. Chem. 211:113014. doi: 10.1016/j.ejmech.2020.113014, PMID: 33218683 PMC7658596

[ref20] García B. Merayo-Lloves J. Martin C. Alcalde I. Quirós L. M. Vazquez F. (2016). Surface proteoglycans as mediators in bacterial pathogens infections. Front. Microbiol. 7:220. doi: 10.3389/fmicb.2016.00220, PMID: 26941735 PMC4764700

[ref21] Giard D. J. Aaronson S. A. Todaro G. J. Arnstein P. Kersey J. H. Dosik H.. (1973). In vitro cultivation of human tumors: establishment of cell lines derived from a series of solid tumors. J. Natl. Cancer Inst. 51, 1417–1423. doi: 10.1093/jnci/51.5.1417, PMID: 4357758

[ref22] Hancock R. E. (1981). Aminoglycoside uptake and mode of action-with special reference to streptomycin and gentamicin. I. Antagonists and mutants. J. Antimicrob. Chemother. 8, 249–276. doi: 10.1093/jac/8.4.249, PMID: 6795174

[ref23] Harrington N. E. Sweeney E. Alav I. Allen F. Moat J. Harrison F. (2021). Antibiotic efficacy testing in an ex vivo model of Pseudomonas aeruginosa and Staphylococcus aureus biofilms in the cystic fibrosis lung. J. Visual. Exp. 167. doi: 10.3791/62187. doi:10.3791/62187 33554970

[ref24] Hawdon N. A. Aval P. S. Barnes R. J. Gravelle S. K. Rosengren J. Khan S.. (2010). Cellular responses of A549 alveolar epithelial cells to serially collected Pseudomonas aeruginosa from cystic fibrosis patients at different stages of pulmonary infection. FEMS Immunol. Med. Microbiol. 59, 207–220. doi: 10.1111/j.1574-695X.2010.00693.x, PMID: 20528926

[ref25] Heydorn A. Nielsen A. T. Hentzer M. Sternberg C. Givskov M. Ersbøll B. K.. (2000). Quantification of biofilm structures by the novel computer program COMSTAT. Microbiology 146, 2395–2407. doi: 10.1099/00221287-146-10-2395, PMID: 11021916

[ref26] Kwon D. H. Lu C. D. (2006). Polyamines increase antibiotic susceptibility in Pseudomonas aeruginosa. Antimicrob. Agents Chemother. 50, 1623–1627. doi: 10.1128/AAC.50.5.1623-1627.2006, PMID: 16641427 PMC1472196

[ref27] Li S. A. Cadelis M. M. Sue K. Blanchet M. Vidal N. Brunel J. M.. (2019). 6-Bromoindolglyoxylamido derivatives as antimicrobial agents and antibiotic enhancers. Bioorg. Med. Chem. 27, 2090–2099. doi: 10.1016/j.bmc.2019.04.004, PMID: 30975502

[ref28] Liao C. Huang X. Wang Q. Yao D. Lu W. (2022). Virulence factors of Pseudomonas aeruginosa and antivirulence strategies to combat its drug resistance. Front. Cell. Infect. Microbiol. 12:926758. doi: 10.3389/fcimb.2022.926758, PMID: 35873152 PMC9299443

[ref29] Liu J. Thorp S. C. (2002). Cell surface heparan sulfate and its roles in assisting viral infections. Med. Res. Rev. 22, 1–25. doi: 10.1002/med.1026, PMID: 11746174

[ref30] Makarov V. A. Popov V. O. (2022). PDSTP is the first drug in class to treat coronavirus infection. Her. Russ. Acad. Sci. 92, 488–490. doi: 10.1134/S1019331622040190, PMID: 36091851 PMC9447965

[ref31] Malinovská L. Thai Le S. Herczeg M. Vašková M. Houser J. Fujdiarová E.. (2019). Synthesis of β-d-galactopyranoside-presenting glycoclusters, investigation of their interactions with Pseudomonas aeruginosa lectin a (PA-IL) and evaluation of their anti-adhesion potential. Biomol. Ther. 9:686. doi: 10.3390/biom9110686, PMID: 31683947 PMC6920806

[ref32] Mann C. M. Markham J. L. (1998). A new method for determining the minimum inhibitory concentration of essential oils. J. Appl. Microbiol. 84, 538–544. doi: 10.1046/j.1365-2672.1998.00379.x 9633651

[ref33] Molina Bertrán S. D. C. Monzote L. Cappoen D. Escalona Arranz J. C. Gordillo Pérez M. J. Rodríguez-Ferreiro A. O.. (2022). Inhibition of bacterial adhesion and biofilm formation by seed-derived ethanol extracts from Persea americana mill. Molecules 27:5009. doi: 10.3390/molecules27155009, PMID: 35956958 PMC9370132

[ref34] Nikolaev Y. A. Tutel'yan A. V. Loiko N. G. Buck J. Sidorenko S. V. Lazareva I.. (2020). The use of 4-hexylresorcinol as antibiotic adjuvant. PLoS One 15:e0239147. doi: 10.1371/journal.pone.0239147, PMID: 32960928 PMC7508414

[ref35] Novoselova E. A. Alimbarova L. M. Monakhova N. S. Lepioshkin A. Y. Ekins S. Makarov V. A. (2020). In vivo activity of pyrimidine-dispirotripiperaziniumin in the male Guinea pig model of genital herpes. J. Virol. Antiviral Res. 9:1. doi: 10.37532/jva.2020.9(1).193

[ref36] Novoselova E. A. Riabova O. B. Leneva I. A. Nesterenko V. G. Bolgarin R. N. Makarov V. A. (2017). Antiretroviral activity of a novel pyrimidyl-di (diazaspiroalkane) derivative. Acta Nat. 9, 105–107. doi: 10.32607/20758251-2017-9-1-105-107, PMID: 28461981 PMC5406667

[ref37] Novoselova E. A. Ryabova O. B. Leneva I. A. Makarov V. A. (2019). Specific antiviral activity of pyrimidinedispirotripiperaziniumalone and in combination with acyclovir on a herpes simplex virus infection model. Pharm. Chem. J. 53, 781–785. doi: 10.1007/s11094-019-02079-9

[ref38] O'Loughlin C. T. Miller L. C. Siryaporn A. Drescher K. Semmelhack M. F. Bassler B. L. (2013). A quorum-sensing inhibitor blocks Pseudomonas aeruginosa virulence and biofilm formation. Proc. Natl. Acad. Sci. USA 110, 17981–17986. doi: 10.1073/pnas.1316981110, PMID: 24143808 PMC3816427

[ref39] Orenti A. Zolin A. Jung A. van Rens J. Fox A. Krasnyk M.. (2022). ECFSPR Annual Report 2020. Available at: https://www.ecfs.eu/projects/ecfs-patient-registry/annual-reports

[ref40] O'Shea R. Moser H. E. (2008). Physicochemical properties of antibacterial compounds: implications for drug discovery. J. Med. Chem. 51, 2871–2878. doi: 10.1021/jm700967e, PMID: 18260614

[ref41] Palmer K. L. Brown S. A. Whiteley M. (2007). Membrane-bound nitrate reductase is required for anaerobic growth in cystic fibrosis sputum. J. Bacteriol. 189, 4449–4455. doi: 10.1128/JB.00162-07, PMID: 17400735 PMC1913347

[ref42] Panda S. K. Buroni S. Swain S. S. Bonacorsi A. da Fonseca Amorim E. A. Kulshrestha M.. (2022). Recent advances to combat ESKAPE pathogens with special reference to essential oils. Front. Microbiol. 13:1029098. doi: 10.3389/fmicb.2022.1029098, PMID: 36560948 PMC9763703

[ref43] Qin S. Xiao W. Zhou C. Pu Q. Deng X. Lan L.. (2022). Pseudomonas aeruginosa: pathogenesis, virulence factors, antibiotic resistance, interaction with host, technology advances and emerging therapeutics. Signal Transduct. Target. Ther. 7:199. doi: 10.1038/s41392-022-01056-1, PMID: 35752612 PMC9233671

[ref44] Sanya D. R. A. Onésime D. Vizzarro G. Jacquier N. (2023). Recent advances in therapeutic targets identification and development of treatment strategies towards Pseudomonas aeruginosa infections. BMC Microbiol. 23:86. doi: 10.1186/s12866-023-02832-x, PMID: 36991325 PMC10060139

[ref45] Schmidtke M. Riabova O. Dahse H. M. Stelzner A. Makarov V. (2002). Synthesis, cytotoxicity and antiviral activity of N, N'-bis-5-nitropyrimidyl derivatives of dispirotripiperazine. Antivir. Res. 55, 117–127. doi: 10.1016/s0166-3542(02)00014-1, PMID: 12076756

[ref46] Schmidtke M. Karger A. Meerbach A. Egerer R. Stelzner A. Makarov V. (2003). Binding of a N, N'-bisheteryl derivative of dispirotripiperazine to heparan sulfate residues on the cell surface specifically prevents infection of viruses from different families. Virology 311, 134–143. doi: 10.1016/s0042-6822(03)00166-1, PMID: 12832211

[ref47] Scoffone V. C. Ryabova O. Makarov V. Iadarola P. Fumagalli M. Fondi M.. (2015). Efflux-mediated resistance to a benzothiadiazol derivative effective against Burkholderia cenocepacia. Front. Microbiol. 6:815. doi: 10.3389/fmicb.2015.00815, PMID: 26300878 PMC4525489

[ref48] Stokes J. M. Mac Nair C. R. Ilyas B. French S. Côté J. P. Bouwman C.. (2017). Pentamidine sensitizes gram-negative pathogens to antibiotics and overcomes acquired colistin resistance. Nat. Microbiol. 2:17028. doi: 10.1038/nmicrobiol.2017.28, PMID: 28263303 PMC5360458

[ref49] Tacconelli E. Carrara E. Savoldi A. Harbarth S. Mendelson M. Monnet D. L.. (2018). Discovery, research, and development of new antibiotics: the WHO priority list of antibiotic-resistant bacteria and tuberculosis. Lancet Infect. Dis. 18, 318–327. doi: 10.1016/S1473-3099(17)30753-3, PMID: 29276051

[ref50] Tuon F. F. Dantas L. R. Suss P. H. Tasca Ribeiro V. S. (2022). Pathogenesis of the Pseudomonas aeruginosa biofilm: a review. Pathogens 11:300. doi: 10.3390/pathogens11030300, PMID: 35335624 PMC8950561

[ref51] Vaara M. Siikanen O. Apajalahti J. Fox J. Frimodt-Møller N. He H.. (2010). A novel polymyxin derivative that lacks the fatty acid tail and carries only three positive charges has strong synergism with agents excluded by the intact outer membrane. Antimicrob. Agents Chemother. 54, 3341–3346. doi: 10.1128/AAC.01439-09, PMID: 20479195 PMC2916294

[ref52] van Tilburg Bernardes E. Charron-Mazenod L. Reading D. J. Reckseidler-Zenteno S. L. Lewenza S. (2017). Exopolysaccharide-repressing small molecules with antibiofilm and antivirulence activity against Pseudomonas aeruginosa. Antimicrob. Agents Chemother. 61, e01997–e01916. doi: 10.1128/AAC.01997-16, PMID: 28223377 PMC5404518

[ref53] Vandecandelaere I. Van Acker H. Coenye T. (2016). A microplate-based system as in vitro model of biofilm growth and quantification. Methods Mol. Biol. 1333, 53–66. doi: 10.1007/978-1-4939-2854-5_5, PMID: 26468099

[ref54] Wang C. H. Hsieh Y. H. Powers Z. M. Kao C. Y. (2020). Defeating antibiotic-resistant bacteria: exploring alternative therapies for a post-antibiotic era. Int. J. Mol. Sci. 21:1061. doi: 10.3390/ijms21031061, PMID: 32033477 PMC7037027

[ref55] Wang G. Brunel J. M. Preusse M. Mozaheb N. Willger S. D. Larrouy-Maumus G.. (2022). The membrane-active polyaminoisoprenyl compound NV716 re-sensitizes Pseudomonas aeruginosa to antibiotics and reduces bacterial virulence. Commun. Biol. 5:871. doi: 10.1038/s42003-022-03836-5, PMID: 36008485 PMC9411590

[ref56] Wojtczak K. Byrne J. P. (2022). Structural considerations for building synthetic glycoconjugates as inhibitors for Pseudomonas aeruginosa lectins. Chem. Med. Chem. 17:e202200081. doi: 10.1002/cmdc.202200081, PMID: 35426976 PMC9321714

[ref57] Wood S. J. Kuzel T. M. Shafikhani S. H. (2023). Pseudomonas aeruginosa: infections, animal modeling, and therapeutics. Cell 12:199. doi: 10.3390/cells12010199, PMID: 36611992 PMC9818774

[ref58] Zhou Y. Huang W. Lei E. Yang A. Li Y. Wen K.. (2022). Cooperative membrane damage as a mechanism for pentamidine-antibiotic mutual sensitization. ACS Chem. Biol. 17, 3178–3190. doi: 10.1021/acschembio.2c00613, PMID: 36269311

