# Supplementary material for: Characterization of the dispirotripiperazine derivative PDSTP as antibiotic adjuvant and antivirulence compound against Pseudomonas aeruginosa
Source: Front Microbiol. 2024 Feb 16;15:1357708. doi: 10.3389/fmicb.2024.1357708 (PMC10904629; doi:10.3389/fmicb.2024.1357708)
Supplement: Supplementary file 1 [file Data_Sheet_1.docx]

**Characterization of the dispirotripiperazine derivative PDSTP as antibiotic adjuvant and antivirulence compound against *Pseudomonas aeruginosa.***

**Andrea Bonacorsi^1^**^†^**, Gabriele Trespidi^1^**^†^**, Viola C. Scoffone^1^, Samuele Irudal^1^, Giulia Barbieri^1^, Olga Riabova^2^, Natalia Monakhova^2^, Vadim Makarov^2^, Silvia Buroni^1*^.**

^1^Department of Biology and Biotechnology "Lazzaro Spallanzani", University of Pavia, Pavia, Italy.

^2^ Research Center of Biotechnology RAS, Moscow, Russia.

*** Correspondence:**Silvia Buroni
silvia.buroni@unipv.it.

^†^These authors contributed equally to this work and share first authorship


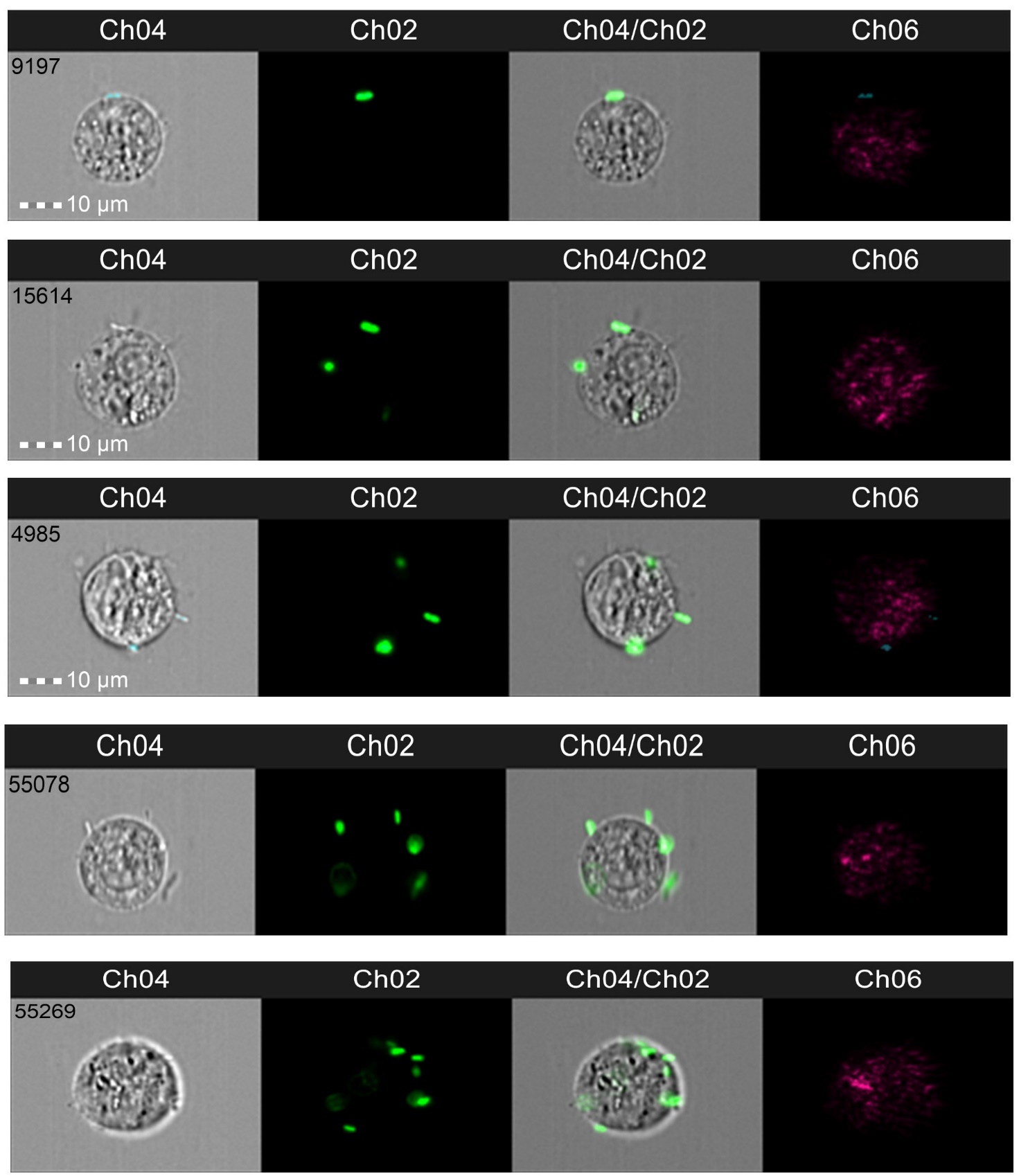
Supplementary Material

**Supplementary Figure 1.** Representative image galleries of A549 cells infected with GFP-expressing *P. aeruginosa* PA01. Ch04: bright field. Ch02: GFP fluorescence (λex/λem = 480-560 nm). Ch04/Ch02: superimposition of bright field and fluorescence channels. Ch06: dark field side scatter. Pictures were acquired at 40x magnification using Amnis^®^ ImageStream^®^X Mk II (Cytek).


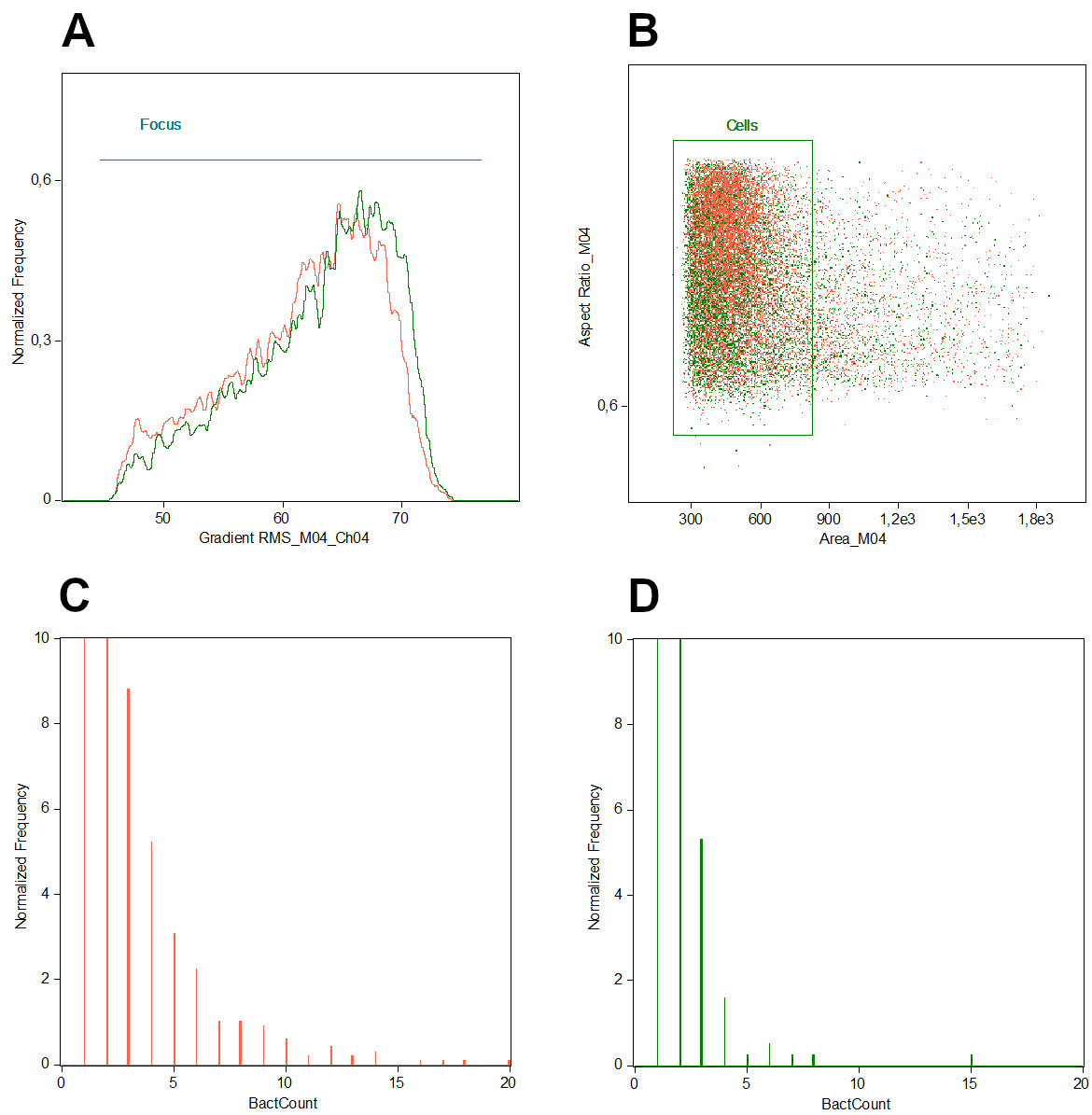


**Supplementary Figure 2.** Counting of GFP-expressing *P. aeruginosa* PA01 cells using Spot Count feature. On focus-cells population defined by GradientRMS feature histogram (A). Single cells population (gated on focus-cells) defined by Area/Aspect ratio features dot plot (B). Untreated sample: histogram of bacteria count using the custom “BactCount” feature (applied to focused, single cells) (C). Treatment with 50 µg/mL of PDSTP sample: histogram of bacteria count using the custom “BactCount” feature (applied to focused, single cells) (D).


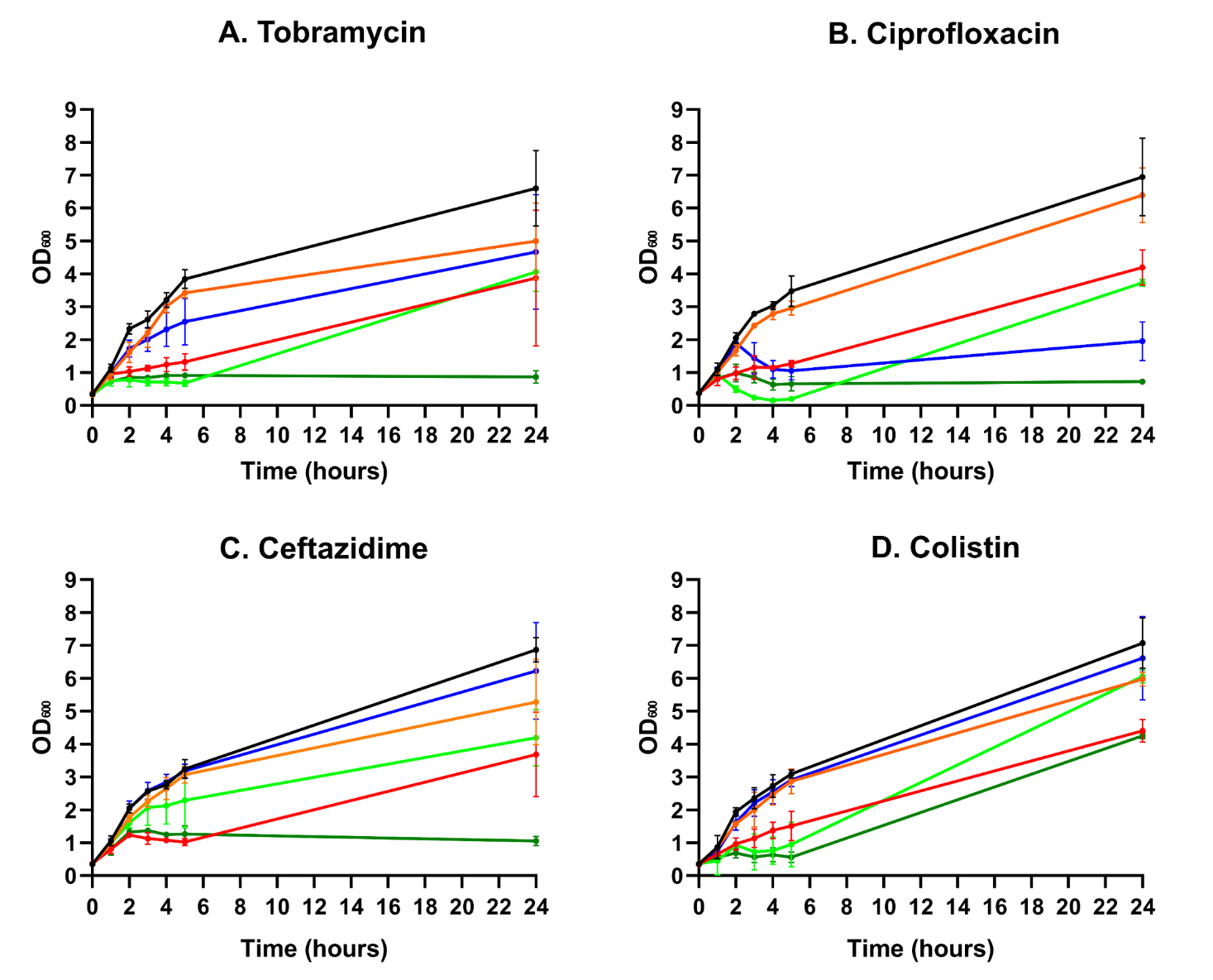
**Supplementary Figure 3.** *P. aeruginosa* PA01 time-killing assays of tobramycin (A), ciprofloxacin (B), ceftazidime (C) and colistin (D) combined with either 50 or 200 µg/mL of PDSTP, represented as variation of optical density at 600 nm. Black line, untreated sample; orange line, treatment with 50 µg/mL of PDSTP; red line, treatment with 200 µg/mL of PDSTP; blue line, treatment with a concentration equal to ½ MIC of the antibiotic; light green line, combination of 50 µg/mL of PDSTP with the antibiotic; dark green line, combination of 200 µg/mL of PDSTP with the antimicrobial.


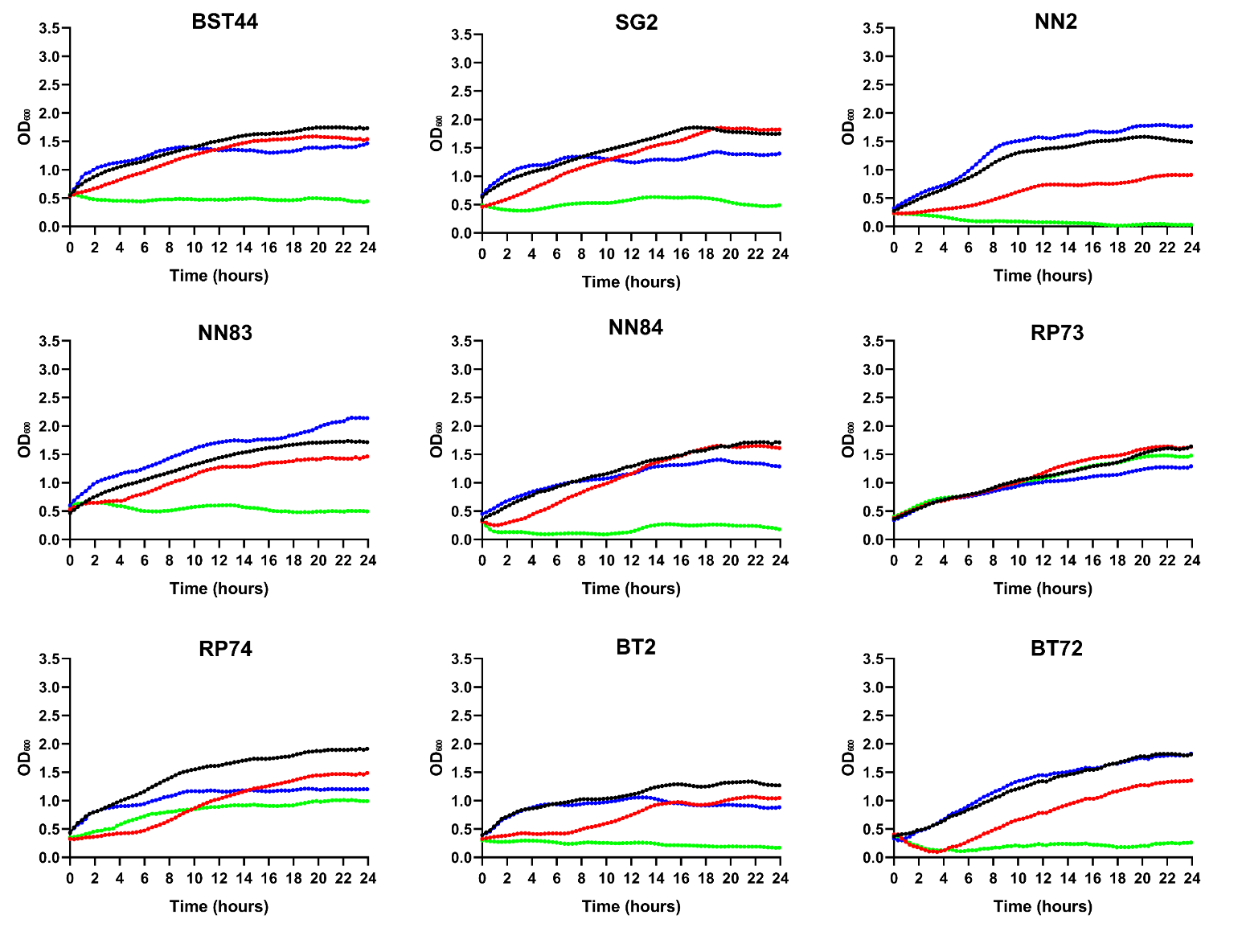
**Supplementary Figure 4.** *P. aeruginosa* CF clinical isolates time-killing assays of ceftazidime combined with PDSTP, represented as variation of optical density at 600 nm. Black line, untreated sample; red line, treatment with sub-inhibitory concentrations of PDSTP; blue line, treatment with sub-inhibitory concentrations of ceftazidime; light green line, combination of PDSTP with the antibiotic.

**
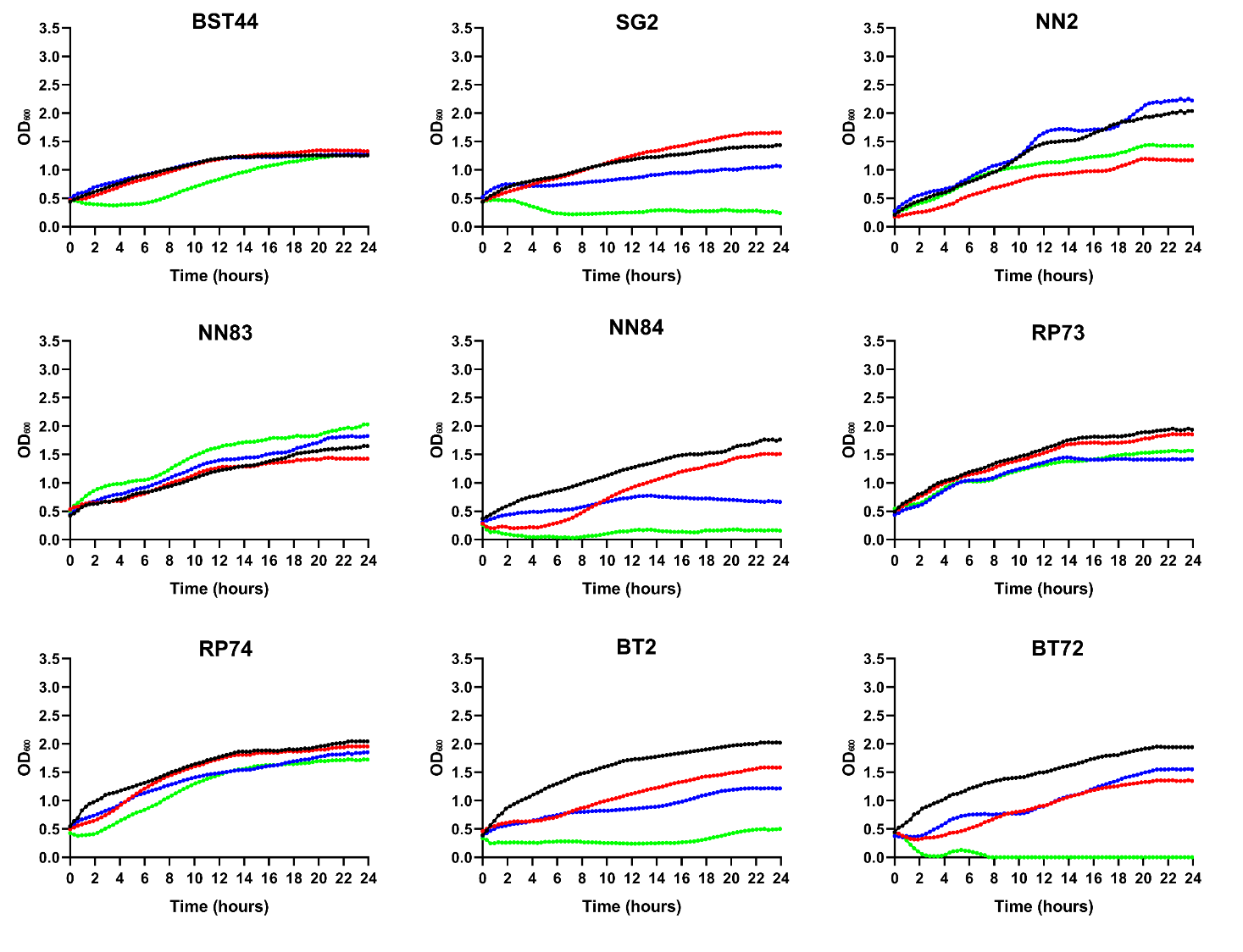
Supplementary Figure 5.** *P. aeruginosa* CF clinical isolates time-killing assays of tobramycin combined with PDSTP, represented as variation of optical density at 600 nm. Black line, untreated sample; red line, treatment with sub-inhibitory concentrations of PDSTP; blue line, treatment with sub-inhibitory concentrations of tobramycin; light green line, combination of PDSTP with the antibiotic.


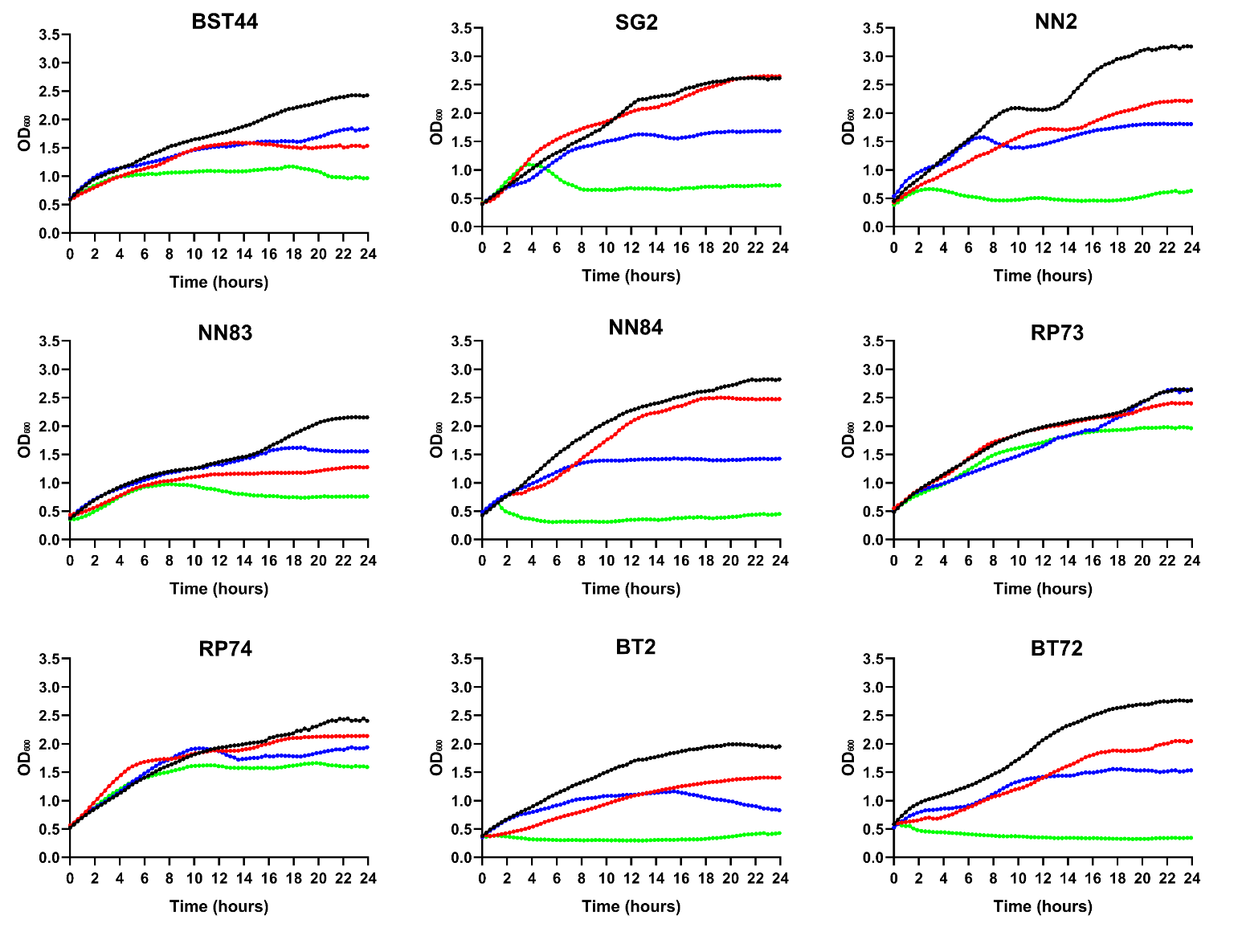
**Supplementary Figure 6.** *P. aeruginosa* CF clinical isolates time-killing assays of ciprofloxacin combined with PDSTP, represented as variation of optical density at 600 nm. Black line, untreated sample; red line, treatment with sub-inhibitory concentrations of PDSTP; blue line, treatment with sub-inhibitory concentrations of ciprofloxacin; light green line, combination of PDSTP with the antibiotic.


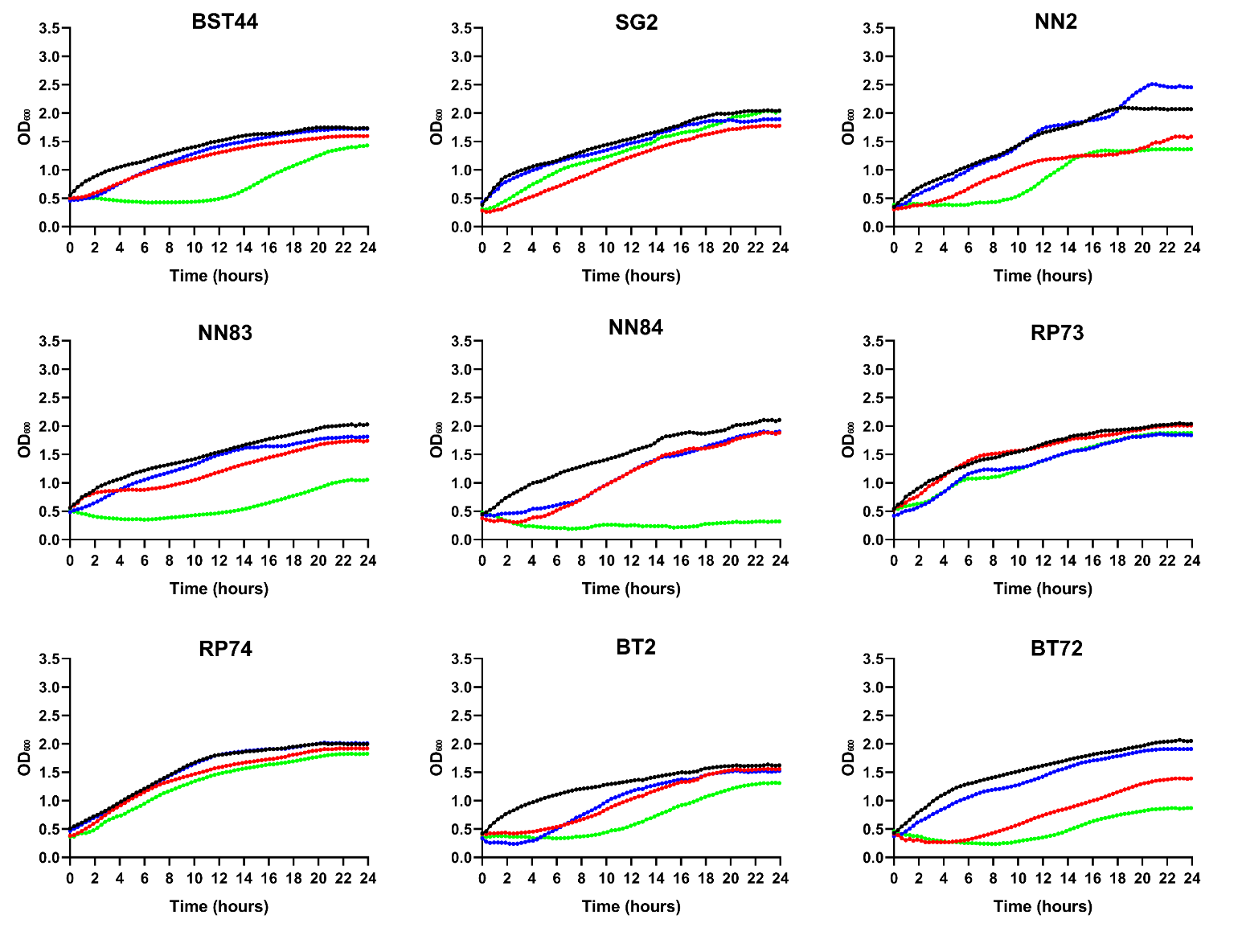
**Supplementary Figure 7.** *P. aeruginosa* CF clinical isolates time-killing assays of colistin combined with PDSTP, represented as variation of optical density at 600 nm. Black line, untreated sample; red line, treatment with sub-inhibitory concentrations of PDSTP; blue line, treatment with a concentration equal to the MIC of colistin; light green line, combination of PDSTP with the antibiotic.


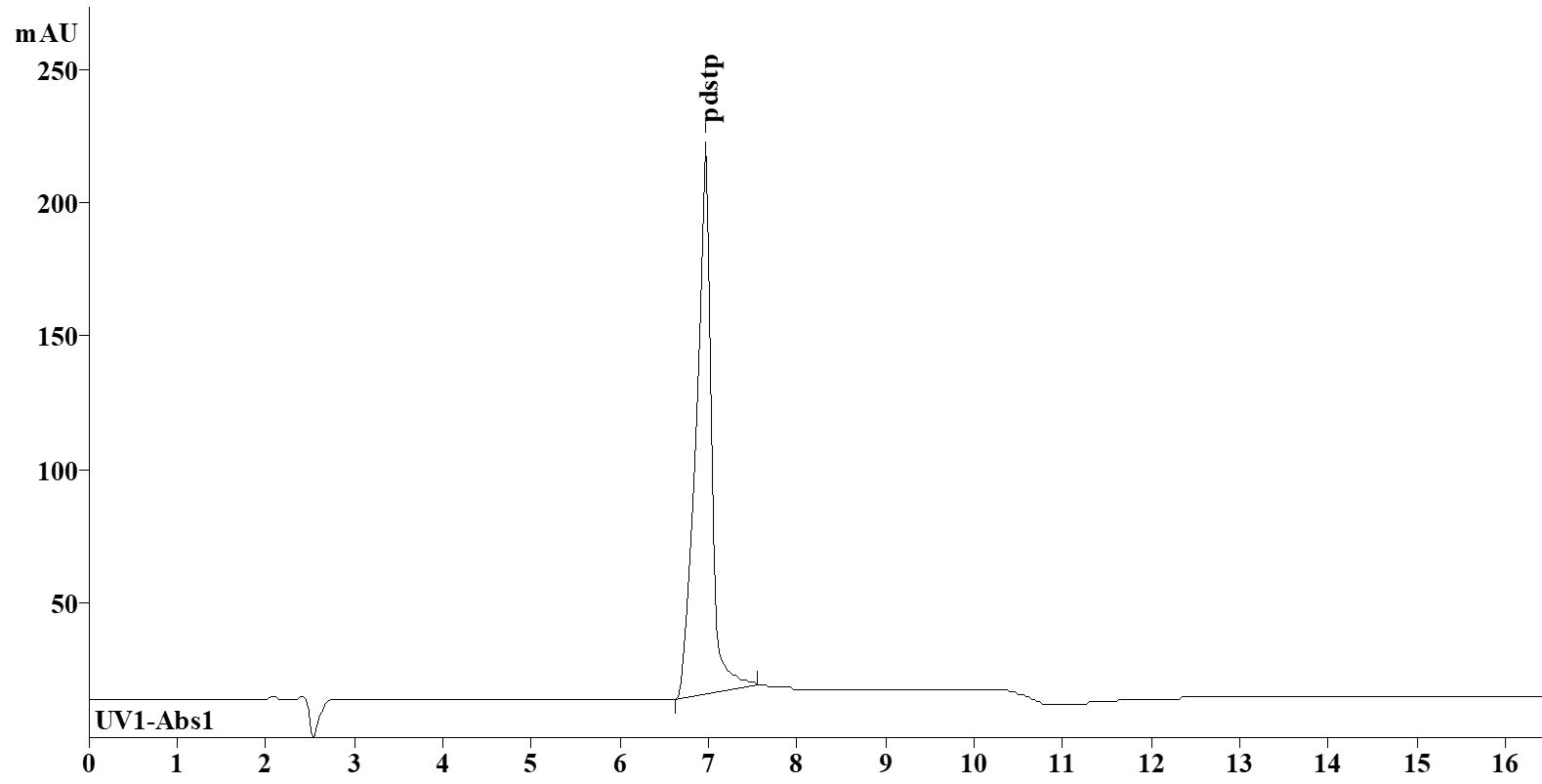


**Supplementary Figure 8.** Purity of PDSTP was analyzed by analytical high-performance liquid chromatography (HPLC) on a LC-20 Prominence HPLC system (Shimadzu, Kyoto, Japan) equipped with a SPD-20A Prominence UV detector at 254 nm. Chromatographic separation was carried out on a Kromasil 60-Diol HPLC column (4.6 × 250 mm, 5 μm; Nouryon, Göteborg, Sweden) at 30 °C, with a sample injection volume of 20 µL. A mobile phase consisting of methanol and DMSO (90:10 v/v), and heptafluorobutyric acid in methanol (0.5:95.5 v/v) was programmed with gradient elution at a flow rate of 1300 µL/min. Data were processed with a LC solution software version 1.25 (Shimadzu).

Peak Results:

| Retention time (min) | Peak height (mAU) | Peak area (mAu·sec) | Peak asymmetry |
| --- | --- | --- | --- |
| 6.955 | 206.39 | 10329 | 0.86 |

**Supplementary Table 1.** Efficacy of the PDSTP-ceftazidime combination treatment against 9 *P. aeruginosa* CF clinical strains, expressed in hours of bacterial growth inhibition.

|  | BST44 | SG2 | NN2 | NN83 | NN84 | RP73 | RP74 | BT2 | BT72 |
| --- | --- | --- | --- | --- | --- | --- | --- | --- | --- |
| Ceftazidime (µg/mL) | 4 | 32 | 2 | 8 | 12 | 16 | 32 | 1 | 0.5 |
| PDSTP (µg/mL) | 200 | 200 | 200 | 100 | 200 | 200 | 200 | 200 | 150 |
| Growth inhibition (hours) | 24 | 24 | 24 | 24 | 24 | None | None | 24 | 24 |

**Supplementary Table 2.** Efficacy of the PDSTP-tobramycin combination treatment against 9 *P. aeruginosa* CF clinical strains, expressed in hours of bacterial growth inhibition.

|  | BST44 | SG2 | NN2 | NN83 | NN84 | RP73 | RP74 | BT2 | BT72 |
| --- | --- | --- | --- | --- | --- | --- | --- | --- | --- |
| Tobramycin (µg/mL) | 2 | 16 | 32 | 32 | 8 | 8 | 1 | 0.75 | 4 |
| PDSTP (µg/mL) | 200 | 200 | 200 | 100 | 200 | 200 | 200 | 200 | 150 |
| Growth inhibition (hours) | 6 | 24 | None | None | 24 | None | None | 24 | 24 |

**Supplementary Table 3.** Efficacy of the PDSTP-ciprofloxacin combination treatment against 9 *P. aeruginosa* CF clinical strains, expressed in hours of bacterial growth inhibition.

|  | BST44 | SG2 | NN2 | NN83 | NN84 | RP73 | RP74 | BT2 | BT72 |
| --- | --- | --- | --- | --- | --- | --- | --- | --- | --- |
| Ciprofloxacin (µg/mL) | 0.0156 | 0.125 | 0.25 | 0.25 | 2 | 0.25 | 1 | 0.0312 | 0.0312 |
| PDSTP (µg/mL) | 200 | 200 | 200 | 100 | 200 | 200 | 200 | 200 | 150 |
| Growth inhibition (hours) | 24* | 24* | 24 | 24* | 24 | None | None | 24 | 24 |

* OD_600_ of the combination treatment remained stably lower than the other conditions for 24 hours but a modest increase of the OD_600_ occurred within the first hours of treatment.

**Supplementary Table 4.** Efficacy of the PDSTP-colistin combination treatment against 9 *P. aeruginosa* CF clinical strains, expressed in hours of bacterial growth inhibition.

|  | BST44 | SG2 | NN2 | NN83 | NN84 | RP73 | RP74 | BT2 | BT72 |
| --- | --- | --- | --- | --- | --- | --- | --- | --- | --- |
| Colistin (µg/mL) | 2 | 0.125 | 0.5 | 1 | 2 | 2 | 0.0625 | 2 | 1 |
| PDSTP (µg/mL) | 200 | 200 | 200 | 100 | 200 | 200 | 200 | 200 | 150 |
| Growth inhibition (hours) | 12 | None | 10 | 12 | 24 | None | None | 10 | 10 |

**Supplementary Table 5.** Minimum inhibitory concentrations in TSB of ceftazidime, tobramycin, ciprofloxacin and colistin against *P. aeruginosa* CF clinical isolates.

|  |  | BST44 | SG2 | NN2 | NN83 | NN84 | RP73 | RP74 | BT2 | BT72 |
| --- | --- | --- | --- | --- | --- | --- | --- | --- | --- | --- |
| MIC (µg/mL) | **Ceftazidime** | 8 | 64 | 8 | 16 | 16 | 16 | 64 | 4 | 8 |
|  | **Tobramycin** | 8 | 16 | >256 | >256 | 16 | 32 | 4 | 1 | 4 |
|  | **Ciprofloxacin** | 0.25 | 0.25 | 1 | 1 | 4 | 1 | 4 | 1 | 0.25 |
|  | **Colistin** | 2 | 0.125 | 0.5 | 1 | 2 | 2 | 0.0625 | 2 | 1 |
